# Preclinical development of anti-CD21 chimeric antigen receptor T cells to treat T cell acute lymphoblastic leukemia

**DOI:** 10.1126/scitranslmed.adr1476

**Published:** 2025-04-16

**Authors:** Nicola Maciocia, Malika Hoekx, Ciaran Acuna, Brandon Wade, Amy Burley, Saumya Ramanayake, Francesco Nannini, Patrycja A. Wawrzyniecka, Thaneswari Karpanasamy, Maria Schuldt, Stephanie Ng, Mathieu Ferrari, Teresa Marafioti, Giuseppe Gritti, Shimobi Onuoha, David O’Connor, Lydia Lee, Marc Mansour, Asim Khwaja, Martin Pule, Paul Maciocia

**Affiliations:** 1 Department of Haematology, Cancer Institute, University College London, 72 Huntley Street, London WC1E6DD, UK; 2 Autolus Ltd., Mediaworks, 191 Wood Lane, White City, London W12 7FP, UK; 3 Department of Haematology, Ospedale Papa Giovanni XXIII, Piazza OMS 1, 24127 Bergamo, Italy

## Abstract

Patients with relapsed/refractory (r/r) T cell acute lymphoblastic leukemia (T-ALL) have a dismal prognosis, highlighting the urgent need for effective therapies. Chimeric antigen receptor (CAR)–T cell approaches targeting pan–T cell antigens may be limited by T cell aplasia and fratricide, necessitating “rescue” allogeneic hematopoietic stem cell transplantation. In this study, we identify CD21, a pan–B cell marker, as a promising target for T-ALL immunotherapy. CD21 is expressed in 50% of T-ALL cases at diagnosis but in fewer than 10% of mature T cells. We observed that CAR-T cells targeting membrane-distal CD21 epitopes were ineffective, likely because of the bulky, glycosylated nature of the antigen. However, when we engineered CAR-T cells to target membrane-proximal CD21 epitopes using an antigen-binding fragment (Fab)–CAR design, we demonstrated robust activity against T-ALL cell lines, primary tumors, and patient-derived xenografts in both in vitro and in vivo models. The enhanced efficacy of this Fab-CAR design was driven by its high stability and reduced surface expression, addressing limitations of traditional CAR constructs. In addition, pharmacological inhibition of the phosphatidylinositol 3-kinase axis up-regulated CD21 expression in T-ALL, further enhancing the potency of anti-CD21 CAR-T cells in vitro and in a patient-derived xenograft in vivo model. This study establishes CD21 as a viable CAR-T target and highlights advances in CAR design for bulky antigens, as well as the potential for pharmacological strategies to augment target expression. Anti-CD21 CAR-T cells represent a promising therapeutic option for improving outcomes for patients with T-ALL.

## Introduction

T cell acute lymphoblastic leukemia (T-ALL) is an aggressive malignancy of T cell precursors, accounting for ~15% of ALL diagnoses in children and 25% in adults (1, 2). Outcomes have been historically poor, but recent advances in treatment have brought them in line with those for B cell acute lymphoblastic leukemia (B-ALL) (3, 4). However, the prognosis for patients with relapsed or refractory (r/r) disease is dismal, with only 6.5% long-term survival in adults and <25% in children (2, 5).

Chimeric antigen receptor (CAR)–T cell therapy is effective for patients with r/r B-ALL, inducing sustained responses in ~40 to 50% of patients (6). Early studies suggest that CAR-T cell therapies may also be useful for patients with T-ALL. However, CAR-T cell targeting of T-ALL is more complex: Analogous approaches to CD19 CAR-T cell therapy, where T cell lineage antigens, such as CD5 and CD7 (7–9), are targeted, carry risks of fratricide and T cell aplasia. This may require additional engineering and possible rescue by hematopoietic stem cell transplantation (HSCT).

Targeting antigens only expressed on T-ALL blasts, without expression on normal T cells, such as TRBC1, CD1a, and CCR9 (10–12), is more desirable. However, this latter approach is limited because only a proportion of T-ALL cases express target-selective antigens. Identification of additional targets is required to maximize the proportion of patients with T-ALL who can benefit from CAR-T cell therapies. Moreover, in B-ALL, antigen-negative escape has emerged as a major mechanism of resistance. Dual-targeting approaches are being explored in clinical trials, and it is likely that analogous strategies will be required for treatment of T-ALL (13, 14).

CD21 (*CR2, complement receptor 2*) is a type 1 transmembrane glycoprotein containing 15 of 16 highly conserved short consensus repeats (SCRs). It is expressed on mature B lymphocytes and follicular dendritic cells (FDCs), where it binds complement within the B cell coreceptor complex alongside CD19, CD81, and CD225 (15, 16). It has no known expression on nonimmune tissues.

Here, we explored CD21 as a promising CAR-T target for treatment of patients with T-ALL and demonstrated that CD21 is expressed in 50% of patients with T-ALL but only in 10% of normal T cells. CD21 is a complex CAR-T target because of its large, flexible, and highly glycosylated extracellular domain. Despite this, we show that anti-CD21 CAR-T cells targeting membrane-proximal epitopes and using an antigen-binding fragment (Fab) antigen-binding domain have robust in vitro and in vivo antitumor activity in multiple T-ALL models. Furthermore, we show that anti-CD21 CAR-T efficacy can be enhanced through phosphatidylinositol 3-kinase (PI3K)–mediated pharmacological up-regulation of CD21 antigen density, representing a promising potential treatment strategy for patients with T-ALL.

## Results

### CD21 is aberrantly expressed in T-ALL

**Fig. 1:**
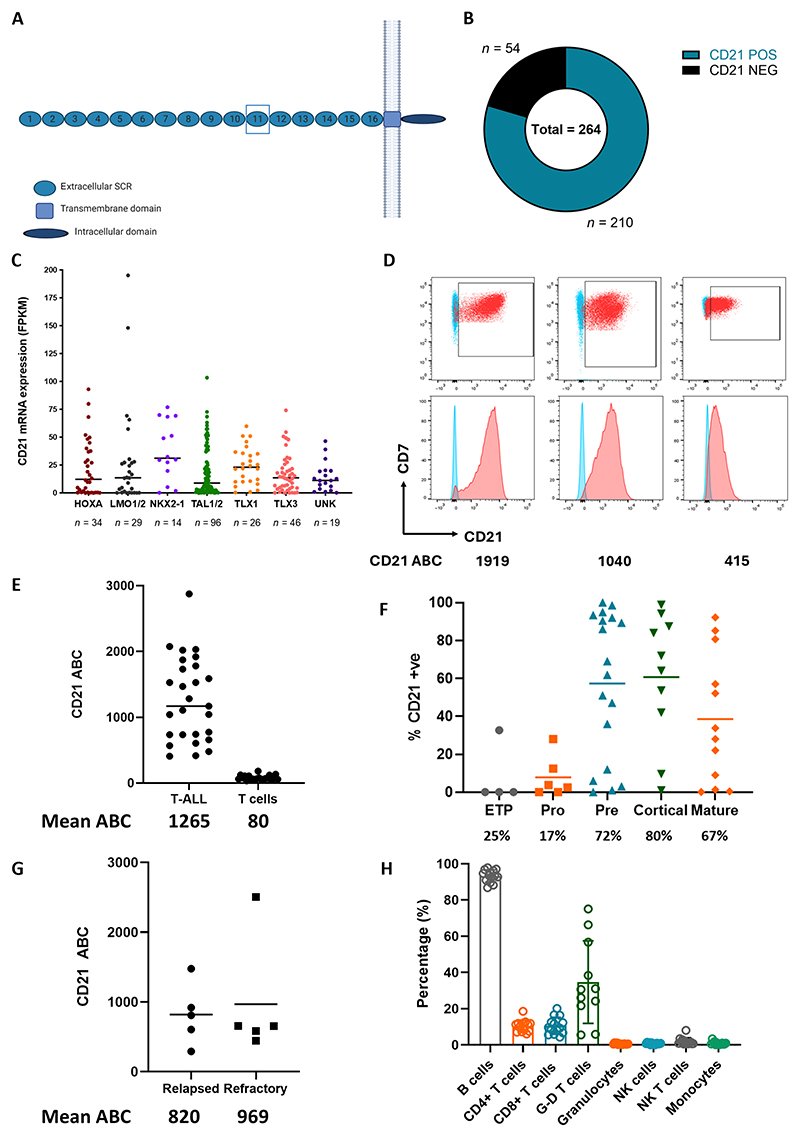
T-ALL–derived cell lines and primary tumors express surface CD21 according to flow cytometry with only limited expression seen on normal T cells. (**A**) Schematic diagram of the structure of CD21—extracellular domain consisting of 15 of 16 highly conserved SCRs. (**B**) *CR2* (CD21) RNA expression in 264 primary T-ALL cases. Data are from St. Jude’s dataset (16). (**C**) *CR2* RNA expression in the same cohort according to molecular subtype. Horizontal bars represent mean value. FPKM, fragments per kilobase of transcript per million mapped reads. (**D**) Example flow plots and histograms of three primary T-ALL samples (red: CD21; blue: isotype control) including ABC of each sample. (**E**) Flow cytometry evaluation of CD21 antigen density (ABC) for CD21-positive diagnostic T-ALL cases, *n* = 27, alongside CD21 density on corresponding normal T cells. Horizontal bar represents median value. (**F**) CD21 expression in diagnostic T-ALL samples by maturation status: ETP–, pro–, pre–, cortical, and mature T-ALL. *n* = 50. Horizontal bars represent mean value. (**G**) ABC of CD21-positive relapsed and refractory T-ALL cases. Horizontal bars represent mean value. (**H**) CD21 expression on PBMCs. Six experimental repeats, *n* = 14 donors. G-D T cells, γδ T cells.

We first aimed to investigate the expression of CD21 (Fig. 1A) across published datasets of T-ALL. CD21 (*CR2*) RNA expression was detected in 80% (210 of 264) of T-ALL cases in a published pediatric cohort (Fig. 1B), across all molecular subtypes (Fig. 1C) (17). In this pediatric dataset (17) and a second adult dataset (UKALL14) (18), *CR2* RNA expression correlated with expression of the *NOTCH1* target *DTX1*, with only limited correlation with *CCR9*, consistent with the known NOTCH control of *CR2* expression (fig. S1, A to D) (12). Next, we evaluated expression across immortalized T-ALL cell lines. Seventy percent (11 of 16) of T-ALL cell lines we tested by flow cytometry were CD21 positive (defined as >20% of cells positive), with a mean antigen density [antibodies bound per cell (ABC)] of 2538 (fig. S2, A and B). Eleven of 13 cell lines with *NOTCH1* mutations expressed CD21, whereas three *NOTCH1* wild-type (WT) cell lines were CD21 negative (fig. S2A).

Next, we evaluated primary patient samples that we collected from the UK CellBank, Great Ormond Street Hospital, or University College London Hospital. Fifty percent (29 of 58) of T-ALL bone marrow samples obtained at diagnosis (43 of 54 for which age data were available were from individuals aged <18 years) expressed CD21. In most positive samples, CD21 was uniformly expressed, with only a few cases showing heterogeneity of expression (Fig. 1D and fig. S3A). The mean ABC of CD21 surface expression on diagnostic samples was 1265 (range 408 to 2875). CD21 was minimally expressed on normal T cells in these samples (>10-fold lower than lymphoblasts, mean of 80 antigens per cell; Fig. 1E).

Next, we evaluated whether CD21 expression varied according to maturation stage in these same patient samples. We found that the highest expression was seen in cortical (80%), pre– (72%), and mature T-ALL (67%), and lesser expression was present in early T cell precursor (ETP)–ALL (25%) and pro–T-ALL (17%) (Fig. 1F). We also evaluated CD21 expression in pediatric and adult cases of r/r T-ALL collected from similar sources. Forty-five percent (5 of 11) of marrow samples obtained from patients with relapsed T-ALL were CD21+ by flow cytometry (mean ABC: 820). Seventeen percent (5 of 30) of samples from patients with primary progressive T-ALL expressed CD21 (mean ABC: 969) (Fig. 1G and fig. S3A).

To evaluate the potential for CD21 CAR-T in a dual-targeting approach, we used flow cytometry to compare CD21 expression with the previously described T-ALL targets CD7, CCR9, and CD1a in 50 of these already described diagnostic T-ALL samples, where extended phenotyping was available. Sixty-nine percent were CCR9+, 57% CD21+, and 20% CD1a+. In this cohort, 14% of cases were CD21+ but CCR9 negative, 26% were CCR9+ but CD21 negative, and 8% were CD1a+ but CD21 negative. Considering coexpression with CD21, 43% of cases were CD21+ CCR9+, whereas only 12% were CD21+ CD1a+. Only 16% of cases did not express either CCR9 or CD21. As expected, 48 of 50 (96%) cases were CD7+ (fig. S4). Thus, dual-targeting anti-CCR9 and anti-CD21 could allow T cell–sparing CAR-T treatment for 84% of patients in this cohort.

### CD21 expression is limited to B cells and a minor fraction of T cells

Next, we examined the expression of CD21 (*CR2*) in normal tissues. Analysis of the Human Protein Atlas showed that *CR2* RNA expression in peripheral blood is limited to B cells and a small fraction of T cells (fig. S5A). No expression of *CR2* on nonlymphoid normal tissues was seen (fig. S5B). To explore expression in thymic subsets, we obtained thymic tissues from children undergoing incidental thymic resection during cardiac surgery. *CR2* was expressed on developing thymocytes: It was highest on immature double-negative cells, before becoming lost in mature double-positive and single-positive subsets (fig. S5C) (19). Next, we used flow cytometry to assess protein expression of CD21 in blood from healthy individuals (*n* = 12). CD21 was highly expressed on B cells but was not detected on granulocytes, natural killer (NK) cells, NK-T cells, or monocytes. CD21 was detected on few αβ T cells with similar expression in CD4 (mean 10% CD21+) and CD8 cells (mean 11% CD21+; Fig. 1H). CD21+ T cells in these healthy donor samples expressed CD21 at low surface density (mean T cell CD21 ABC: 316), 10-fold lower than B cells and fourfold lower than T lymphoblasts. CD21 expression was highest in γδ and naive T cell subsets (mean 15 and 20%, respectively), still at low density (mean naïve T cell ABC: 316; mean γδ T cell ABC: 335; and mean B cell ABC: 4250) (fig. S6, A and B). CD21 was not up-regulated on activated T cells and showed no correlation with the activation marker HLA-DR (fig. S6, C and D). These experiments suggested that CD21 is a T-ALL–selective immunotherapy target without expression on most T cells or other essential tissues.

### CARs targeting CD21 membrane-distal epitopes are ineffective

**Fig. 2:**
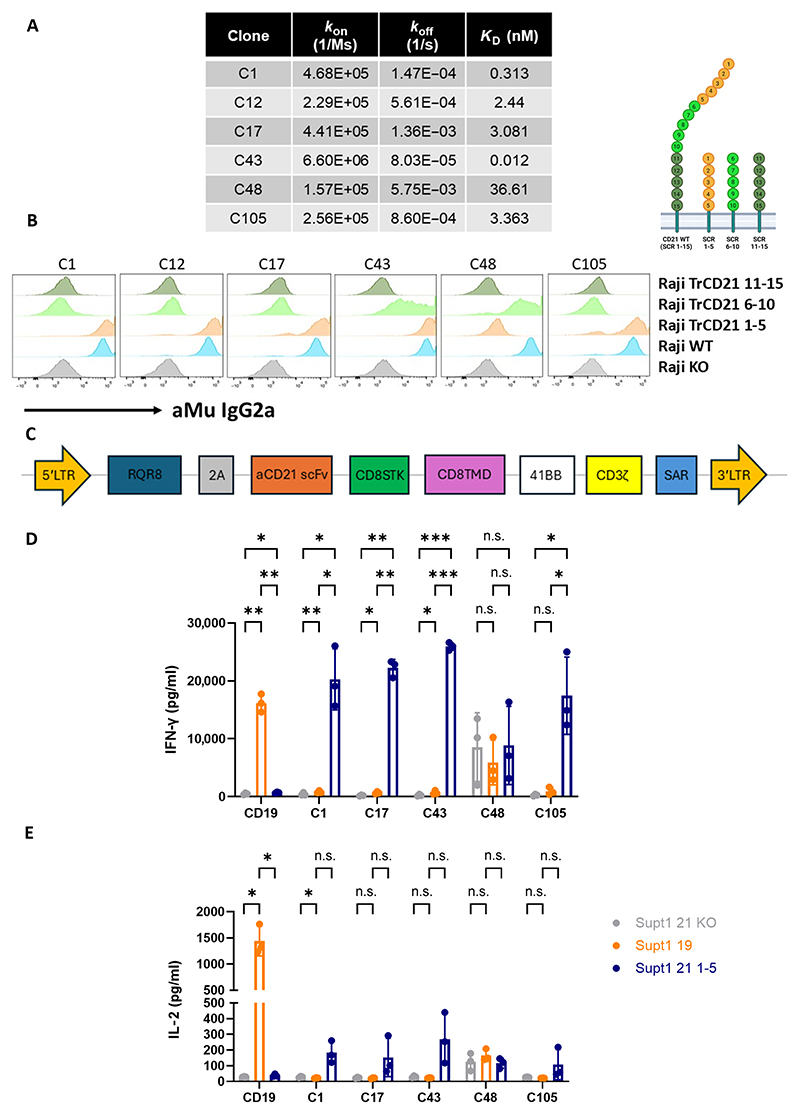
CAR-T cells targeting CD21 distal epitopes are not functional in vitro. (**A**) Surface plasmon resonance (SPR) measurements for six anti-CD21 scFvs. *k*_on_ (*k*_a_), association rate constant; *k*_off_ (*k*_d_), dissociation rate constant; *K*_D_ (*k*_d_/*k*_a_), equilibrium dissociation constant. (**B**) Epitope mapping of anti-CD21 scFvs against Raji CD21 mutant cell lines. Dark green: Raji 21SCR11 to 15 (five most membrane-proximal domains); light green: Raji 21SCR6 to 10 (five middle domains); orange: Raji 21SCR1 to 5 (five most membrane-distal domains); blue: Raji WT [naturally expresses full-length CD21 (SCRs 1 to 15)]; gray, Raji 21 KO (engineered to eliminate surface expression of CD21). (**C**) The SFG retroviral vector with second-generation chimeric receptor architecture [CD8 stalk spacer (STK), CD8 TMD, 41BB co-stimulatory domain, and CD3ζ signaling domain]. RQR8, marker/suicide gene; LTR, long terminal repeat. (**D**) IFN-γ and (**E**) IL-2 secretion by ELISA from NT, anti-CD19 and anti-CD21 CAR-T cells against SUPT1 19 (CD21 and CD19 positive), SUPT1 21KO (CD19 and CD21 negative), and SUPT1 21 SCRs 1 to 5 after 48-hour coculture. *n* = 3, one experiment. Statistical comparisons by repeated measures two-way ANOVA. *P = <0.05; **P = <0.01; ***P = <0.001; n.s., not significant.

Next, we attempted to make CAR-T cells targeting CD21. Six anti-CD21 single-chain variable fragments (scFvs) were selected from an immune phage library, generated by immunizing Wistar rats with the full-length CD21 ectodomain (12). All six scFvs exhibited nanomolar binding affinities (Fig. 2A). To map their binding epitopes on CD21, we engineered variants of Raji WT cells, a B cell lymphoma line that natively expresses CD21. Using CRISPR-Cas9, we deleted CD21 expression to create Raji-CD21KO cells and then retrovirally transduced these with truncated CD21 constructs, each expressing one of three variants: the five membrane-proximal SCRs (Raji TrCD21 SCRs 11 to 15), the five middle SCRs (Raji TrCD21 SCRs 6 to 10), or the five membrane-distal SCRs (Raji TrCD21 SCRs 1 to 5) (Fig. 2B). All six scFvs bound full-length CD21 on Raji WT cells, with five of six recognizing epitopes within the membrane-distal SCRs 1 to 5, whereas none bound to the membrane-proximal regions (Fig. 2B).

From these scFvs, second-generation CARs were constructed (Fig. 2C), incorporating a CD8 stalk spacer, a CD8 transmembrane domain (TMD), and a 41BB-z endodomain. To test their efficacy, SUPT1 cells, a T-ALL cell line natively expressing CD21, were retro-virally transduced to express CD19, generating CD19+ SUPT1 cells (CD21 ABC: 4535; CD19 ABC: 84,794). This setup enabled bench-marking of CAR performance against a control anti-CD19 CAR. To generate negative control cells, CD21 expression was separately deleted in SUPT1 cells using CRISPR-Cas9, creating SUPT1-CD21KO cells (CD21 ABC: 0; CD19 ABC: 0). Initial CAR-T constructs showed no in vitro functionality, with no antigen-specific secretion of interferon-γ (IFN-γ) (Fig. 2D) or interleukin-2 (IL-2) (Fig. 2E) in coculture with CD19+ SUPT1 cells (orange). Testing two CAR constructs (C43 and C48) with either a short immunoglobulin G1 (IgG1) hinge spacer or a long IgG1 CH2-CH3 spacer (mutated to prevent Fc binding) failed to improve function (fig. S7).

To investigate the underlying cause of poor efficacy, we engineered SUPT1-CD21KO cells to express a truncated CD21 variant limited to the membrane-distal SCRs 1 to 5 (SupT1-21 SCRs 1 to 5; CD21 ABC: 3948; CD19 ABC: 0). This configuration was expected to create a shorter cell-to-cell distance and potentially improve immunological synapse formation. The same CARs demonstrated antigen-specific cytokine secretion only in coculture with SupT1-21 SCR 1 to 5 cells (blue), which could not be attributed to higher CD21 antigen density compared with CD19^+^ SUPT1 cells (Fig. 2E). These results suggest that the membrane-distal location or accessibility of the epitope was responsible for the poor in vitro functionality observed with these initial constructs.

### CARs targeting CD21 membrane-proximal epitopes show improved in vitro function

To optimize CAR signaling, an immune library was generated from Wistar rats immunized with truncated CD21 (membrane-proximal domains, SCRs 11 to 15). Sixteen scFvs with nanomolar binding affinities were identified through biopanning followed by manual screening (NM1/NM2) or PacBio next-generation sequencing of the panned library (Ph-derived) (fig. S8A) (20). One scFv (H1) was derived from a hybridoma generated from the same rats. All but one scFv bound to the five most membrane-proximal domains on CD21 (Raji TrCD21 SCRs 11 to 15) (fig. S8B). A further scFv was derived from the mouse anti-CD21 antibody clone Bu32, which bound to SCRs 11 to 15 with a higher binding affinity than any phage display–derived scFv (fig. S8C). Binder diversity was relatively limited with each of the 16 scFvs belonging to one of nine highly similar heavy-chain CDR (complementarity-determining region) 3 families (fig. S9).

All scFvs were cloned into the same CD8STK-CD8TMD-41BBz CAR architecture as previously (Fig. 2C). Here, four CARs (NM2, Ph9, Ph10, and Ph12) had CD21-specific cytotoxicity (fig. S10A). NM2, Ph9, and Ph10 had similar VH (variable heavy domain) CDR3s, suggesting a similar binding epitope (fig. S9). Multiple CARs triggered in vitro IFN-γ secretion against SUPT1-WT (CD21+); however, high basal IFN-γ secretion in coculture with the SUPT1-21KO (CD21-negative) cell line was also present (fig. S10B). No CD21-specific IL-2 secretion was observed (fig. S10C). We confirmed that several aCD21 CAR-T cells secreted high amounts of IFN-γ even when cultured in the absence of targets (fig. S10D). These results suggested to us that the high basal IFN-γ secretion seen was due to ligand-independent tonic signaling.

### Anti-CD21 Fab-CARs exhibit CD21-specific cytotoxicity and cytokine secretion

**Fig. 3:**
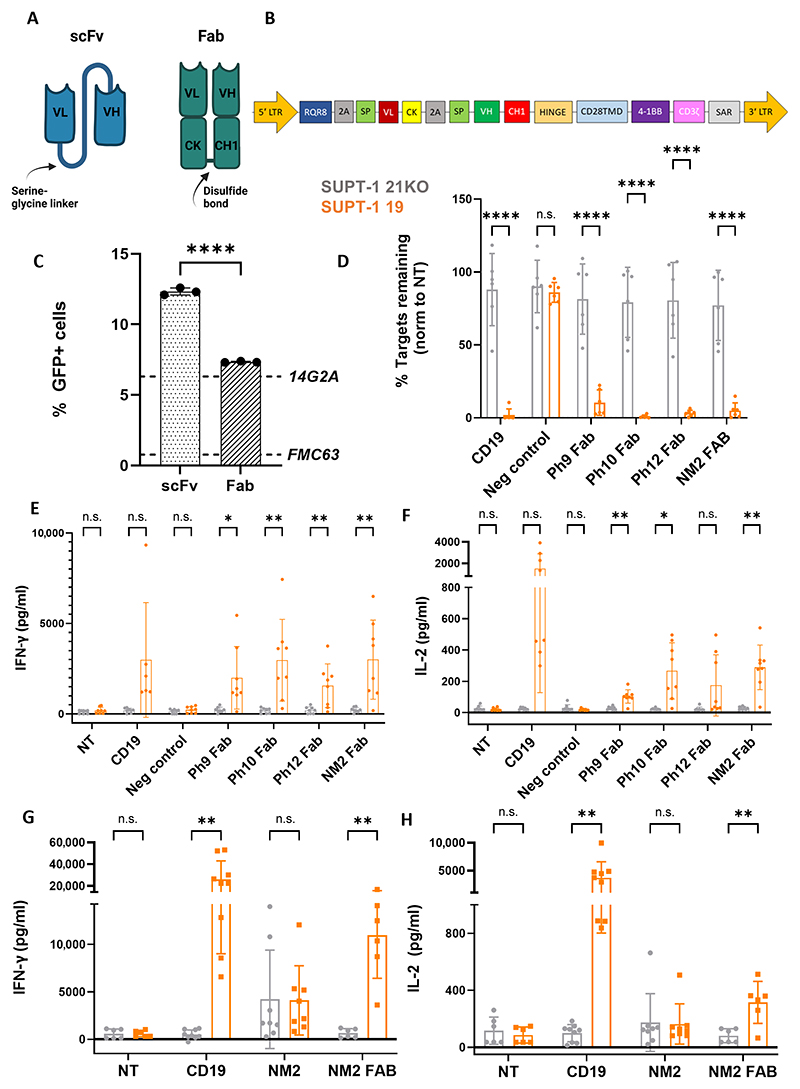
A Fab-CAR architecture reduces tonic signaling and enhances in vitro cytotoxicity. (**A**) Schematic of Fab antigen-binding domain compared with scFv. (**B**) Schematic of anti-CD21 Fab-CARs [SFG retroviral vector with second-generation CAR architecture (hinge spacer, CD28 TMD, 41BB costimulatory domain, and CD3ζ signaling domain)]. 2A, 2A peptide; SP, signal peptide; CK, constant kappa domain; CH1, constant heavy domain 1; SAR, scaffold attachment region. (**C**) NM2 scFv-CARs and Fab-CARs expressed in a Jurkat_NFAT-GFP-CD21KO reporter cell line. Percentage of GFP expression by flow cytometry on day 7 is reported. *n* = 3. Dashed lines represent positive (14G2A) and negative (FMC63) controls. Analyzed by unpaired *t* test. ****P = <0.0001. (**D**) Cytotoxicity (*n* = 6) and (**E**) IFN-γ (*n* = 8) and (**F**) IL-2 (*n* = 8) secretion by NM2, Ph9, Ph10, and Ph12 Fab-CARs by ELISA after 48-hour coculture with SUPT1-19 (orange) and SupT1-21KO (gray) cells. E:T ratio = 1:8. Comparisons made using two-way ANOVA. (**G**) IFN-γ and (**H**) IL-2 secretion from NM2 Fab-CARs and NM2 scFv-CARs after 48-hour coculture against SUPT1-21KO (gray) and SUPT1-19 (orange). NM2, *n* = 9 and NM2, Fab *n* = 6. Three experimental repeats. E:T ratio = 1:4. Pairwise comparisons by two-way ANOVA. *P = <0.05; **P = <0.01; ****P = <0.0001; n.s., not significant.

In an attempt to improve CAR function, selected scFvs recognizing membrane-proximal epitopes were reformatted into 41BBz Fab CARs using a bicistronic vector to express anti-CD21 VH fused to the human IgG1 hinge and CD28 TMD/41BBz endodomain and anti-CD21 VL (variable light domain) fused to the Igκ constant region (Fig. 3, A and B). We hypothesized that this backbone would provide greater CAR stability and efficacy. To further investigate tonic signaling and the effect of Fab architecture upon this, we first expressed both NM2 scFv-CAR and NM2 Fab-CAR in a CD21 knockout (KO) Jurkat NFAT (nuclear factor of activated T cells) reporter cell line. The scFv-CAR showed high ligand-independent signaling, which was significantly reduced with Fab architecture (*P* = <0.0001) (Fig. 3C). We then selected four scFv-CARs (NM2, Ph9, Ph10, and Ph12) that had not only anti-CD21 cytotoxicity but also basal cytokine secretion (fig. S10, B and D), cloned their Fab-CAR counterparts, and then expressed these constructs in T cells. In a 7-day tonic signaling assay where CAR-T cells were plated without target cells, the antigen-independent expansion noted for all scFv-CARs was significantly reduced in three of four Fab-CARs (NM2: *P* = 0.0420; Ph10: *P* = 0.0101; Ph12: *P* = 0.0367) (fig. S11A). Ph9 Fab-CAR also had significantly reduced antigen-independent proliferation (*P* = 0.0138) and expression of the activation/exhaustion marker LAG3 (*P* = 0.0011) (fig. S11C). Two Fab-CARs (NM2: *P* = 0.0003; Ph9: *P* = <0.0001) also had significantly reduced basal IFN-γ secretion (fig. S11D). No difference was seen in T cell differentiation between scFv-CARs and Fab-CARs (fig. S11E).

We next evaluated the efficacy of these four Fab-CARs against CD21-positive and CD21-negative T-ALL cell lines. All demonstrated CD21-specific cytotoxicity and cytokine secretion (Fig. 3, D to F), with NM2 Fab-CAR showing the greatest potency across multiple donors (Fig. 3, G and H). As expected, the cytotoxicity and cytokine secretion of the NM2 Fab-CAR were lower than those of the control anti-CD19 CAR, given the 20-fold higher density of CD19 on SUPT1-19 cells (CD19 ABC: 84,794) compared with CD21 (CD21 ABC: 4535). The potency of the NM2 Fab-CAR was further confirmed in experiments using SUPT1-WT cells, which are CD19 negative (fig. S12, A and B).

### Fab CARs had lower surface expression and enhanced stability

**Fig. 4:**
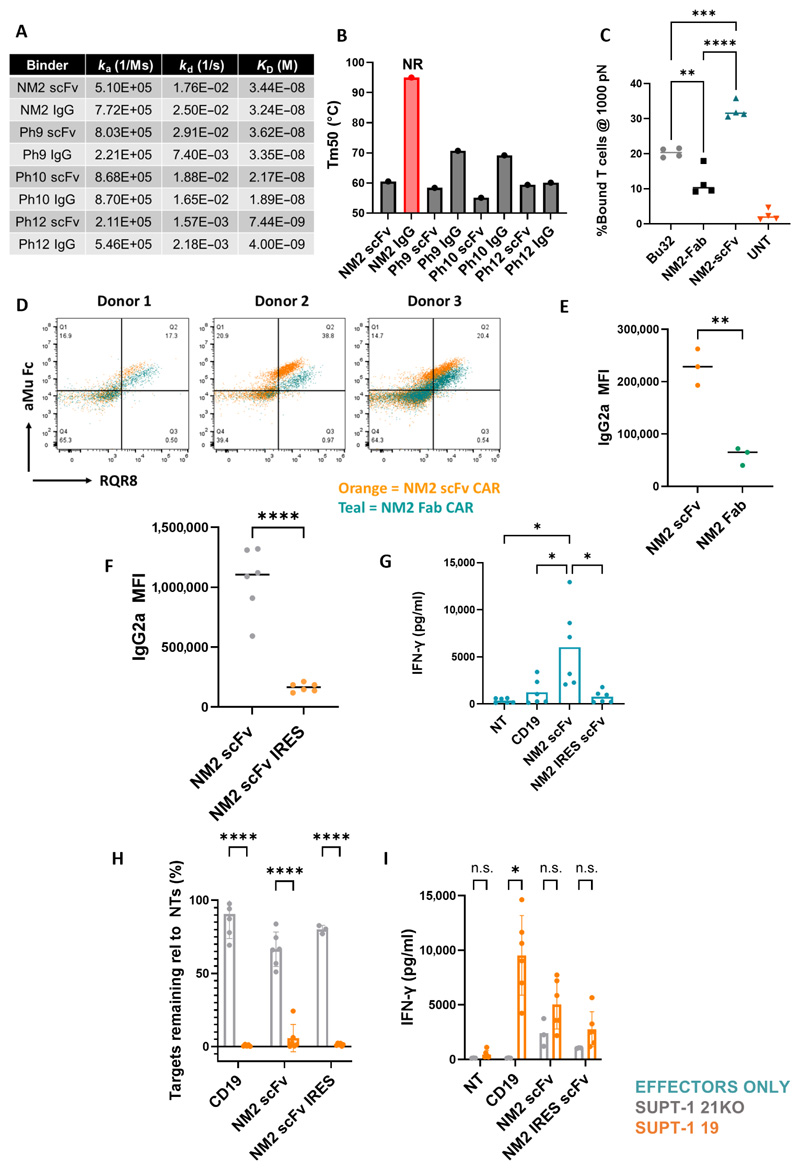
CD21 Fab CARs show lower surface expression and enhanced stability compared with scFv-CARs. (**A**) Binding kinetics of Ph9, Ph10, Ph12, and NM2 scFvs and Fab (IgGs) by SPR. (**B**) Thermal stability of Ph9, Ph10, Ph12, and NM2 scFvs and Fab binders (IgGs) by differential scanning fluorimetry. Tm1/Tm50 = 50% protein unfolding corresponding to variable domain unfolding; Tm50 for NM2 IgG was not reached because the protein had not completely unfolded by 95°C. Mean of two experimental repeats. NR, not reached. (**C**) Immune synapse-binding avidity to MOLT4 WT (CD21+) assessed by acoustic force microfluidic microscopy. Experiment represents one donor CAR-T cells and untransduced (UNT) control on each of the four separate chips. (**D**) NM2 scFv versus Fab CAR21 surface expression donor T cells. *n* = 3. Representative data are from one experiment repeated with the same results. (**E**) Median IgG2a MFI between NM2 scFv-CAR (orange) and NM2 Fab-CAR (teal). *n* = 3. Compared using unpaired *t* test. (**F**) Median IgG2a MFI between NM2 scFv-CAR (gray) and NM2 IRES scFv-CAR (orange). Compared using unpaired *t* test; *P* < 0.0001; *n* = 6. (**G**) Basal IFN-γ secretion (turquoise), (**H**) cytotoxicity, and (**I**) IFN-γ secretion of NM2 scFv-CAR and NM2 IRES scFv-CAR against SUPT1-19 (orange) and against SUPT1-21KO (gray) after 48-hour coculture. 1:4 E:T ratio. Compared using two-way ANOVA. *P = <0.05; **P = <0.01; ***P = <0.001; ****P = <0.0001; n.s., not significant.

We next investigated multiple engineering approaches to enhance the potency of anti-CD21 CAR-T cells and reduce tonic signaling. Initially, we hypothesized that phage display–derived scFvs might inherently promote tonic signaling. However, CARs generated from the high-affinity, proximal-binding antibody Bu32 (*K*_D_: 2.46 × 10^−10^), derived from a mouse hybridoma, also showed high nonspecific IFN-γ secretion and were nonfunctional in both scFv-CAR and Fab-CAR formats (fig. S12, C and D). Next, we examined whether high tonic signaling was linked to overexpression from the SFG gammaretroviral vector. However, switching the NM2 scFv-CAR to a lentiviral vector (pCCL) with an EF1a promoter did not improve cytotoxicity, cytokine secretion, or tonic signaling (fig. S12, E and F). We then explored construct-specific reasons for the improved performance of the NM2 Fab-IgG1-CD28TMD-41BBz (NM2 Fab) CAR. Using whole murine IgG2a, where the binder is naturally in a Fab format, NM2, Ph9, Ph10, and Ph12 and their corresponding scFv-Fc binders had equivalent binding characteristics (Fig. 4A). We also evaluated thermal stability of binders and showed that our lead candidate NM2 Fab binder was uniquely stable, remaining folded up to 95°C (Fig. 4B). In addition, NM2 Fab-CAR showed significantly lower cell-to-cell avidity than NM2 scFv-CAR (*P* = <0.0001) or Bu32 scFv-CAR (*P* = 0.0028) (Fig. 4C).

The impact of CAR surface expression density was also investigated. NM2 Fab-CARs had a lower surface expression than scFv-based CARs, regardless of whether lentiviral or gammaretroviral expression systems were used (Fig. 4, D and E, and fig. S12E). To explore the functional impact of reduced CAR expression further, we generated NM2 scFv-CAR constructs with an upstream internal ribosome entry site (IRES), resulting in significantly reduced CAR surface expression, *P* = <0.0001 (Fig. 4F). This was associated with significantly decreased basal cytokine secretion when CARs were plated alone, *P* = 0.0465 (Fig. 4G). However, although CD21-specific cytotoxicity was comparable between IRES-modified NM2 scFv-CAR and NM2 scFv-CAR (Fig. 4H), IRES NM2 scFv-CAR had less specific cytokine secretion (Fig. 4I). Thus, reduced surface expression could not fully explain the enhanced potency of NM2 Fab-CAR.

We also evaluated the role of the spacer and TMDs in potency and tonic signaling. The NM2 Fab-CAR used a human IgG1 hinge spacer and CD28 TMD, whereas initial scFv-CARs used a CD8 spacer and CD8 TMD. NM2 CAR constructs incorporating either scFv or Fab architecture and varying spacer/TMD combinations were assessed. Both NM2 scFv-CARs were poorly effective: NM2 scFv-8-8 exhibited low cytotoxicity against SUPT1-19 targets, and NM2 scFv-H-28 showed nonspecific killing of SUPT1-CD21KO cells (fig. S13A). These scFv-CARs also demonstrated high basal IFN-γ secretion (fig. S13B) and elevated surface expression compared with Fab-CARs (fig. S13C). Among Fab-CARs, NM2 Fab-8-8 displayed specific cytotoxicity but reduced IFN-γ and undetectable IL-2 secretion. NM2 Fab-H-28 achieved the best performance, demonstrating specific cytotoxicity and robust secretion of both IFN-γ and IL-2 (fig. S13D). Our findings suggest that the optimal performance of NM2 Fab-H-28 CAR-T cells is attributed to the targeting of a membrane-proximal epitope, enhanced binder stability, and reduced CAR surface density.

### NM2 CD21 Fab CAR is effective against CD21 low-density T-ALL cell lines and PDX

**Fig. 5:**
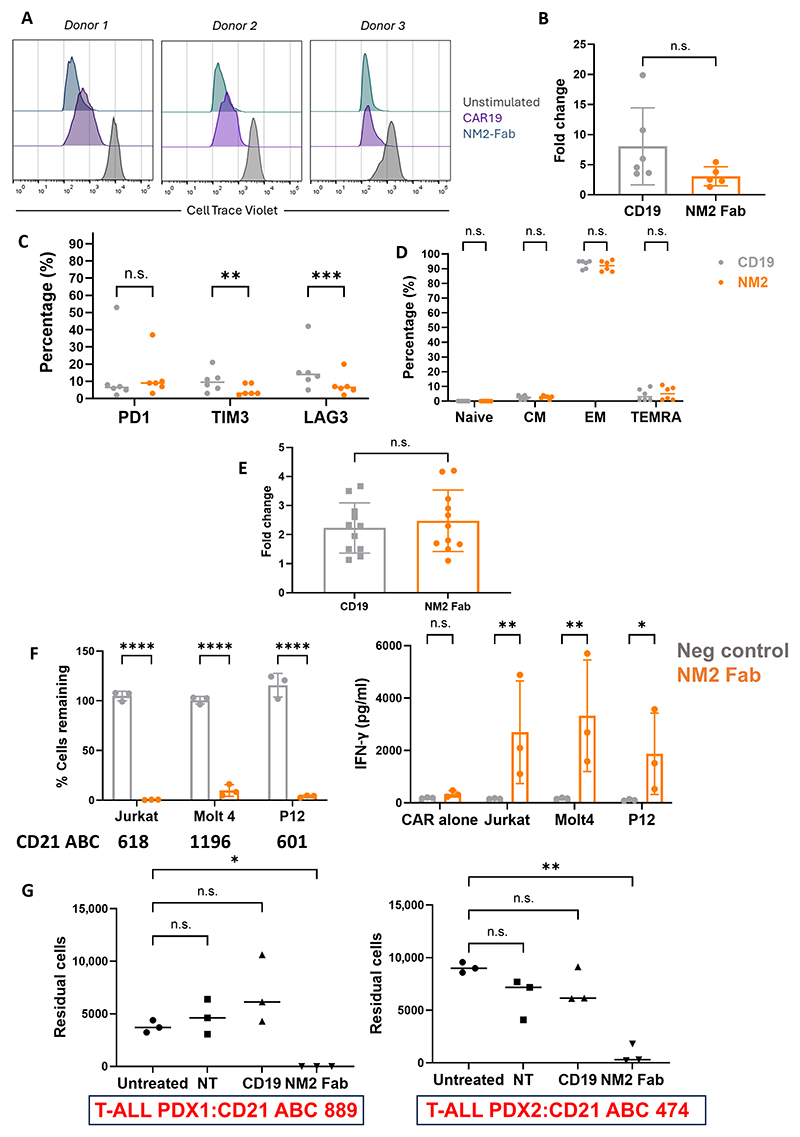
In vitro functional evaluation of NM2 Fab anti-CD21 CAR-T cells. (**A**) Representative histograms of CTV MFI on day 7 of proliferation assay after 1:2 coculture with irradiated CD21+ SUPT1 cells. *n* = 3. (**B**) Day 0 to 7 fold increase in CAR-T numbers in proliferation assay comparing NM2 Fab with CD19 CAR-T, *n* = 6. (**C**) Exhaustion and (**D**) differentiation marker expression on day 7 (NM2 Fab and CD19 CAR-T). Data were analyzed by two-way ANOVA. *n* = 6. (**E**) Fold change in cell numbers pre– and post–CAR transduction between NM2 Fab and CD19 CAR-T. *n* = 11, four experimental repeats. Comparisons made using a paired *t* test. (**F**) Forty-eight hours of cytotoxicity (left) and IFN-γ secretion (right) by ELISA of NM2 Fab-CAR (orange) and Ph2 negative control CAR (gray) against three CD21–low density cell lines, including IFN-γ secretion from CARs plated without targets. 1:4 E:T ratio. Comparisons made using two-way ANOVA. *n* = 3 donors from one experimental repeat. (**G**) Residual cells from two CD21-low PDX T-ALL samples after 72-hour coculture with allogeneic NM2 Fab-CAR T cells, CAR19 T cells, and NT cells. 1:1 E:T ratio, *n* = 3 donors. Comparisons made using paired *t* test. *P = <0.05; **P = <0.01; ***P = < 0.001; ****P = <0.0001; n.s., not significant.

NM2 Fab-IgG1-CD28TMD-41BBz CAR, hereafter referred to as simply NM2 Fab-CAR, was selected as our lead candidate for further evaluation. NM2 Fab-CAR-T cells proliferated (Fig. 5, A and B) with no difference in expansion, exhaustion, or differentiation compared to control anti-CD19 CAR-T cells (CAR19) (Fig. 5, C and D) in a 7-day proliferation assay with irradiated CD21+ SUPT1 cells. NM2 Fab-CAR-T cells were CD21 negative (fig. S14A) and expanded after transduction comparably to CAR19 (Fig. 5E), indicating limited fratricide of CD21-positive T cells. NM2 Fab-CAR showed CD21-specific cytotoxicity and IFN-γ secretion against three CD21–low density T-ALL cell lines: Jurkat (ABC: 618), MOLT4 (ABC: 1196), and P12 (ABC: 601) (Fig. 5F). Furthermore, when cocultured with two CD21-low (474 and 889 CD21 ABC, respectively) patient-derived xenograft (PDX) models of T-ALL (PDX1 and PDX2), NM2 Fab-CAR-T specifically eliminated both T-ALL lymphoblast populations (Fig. 5G). As expected, NM2 Fab-CAR-T cells were cytotoxic to B cells but not to normal T cells when cocultured with autologous peripheral blood mononuclear cells (PBMCs) (fig. S14B). Moreover, when NM2 Fab-CAR-T cells were cocultured with and without “spiked-in” normal mature autologous B cells, which express higher amounts of CD21 than T-ALL blasts, there was no significant attenuation of anti-CD21 cytotoxicity (fig. S14C).

### NM2 CD21 Fab CARs exhibit antileukemic activity in vivo

**Fig. 6:**
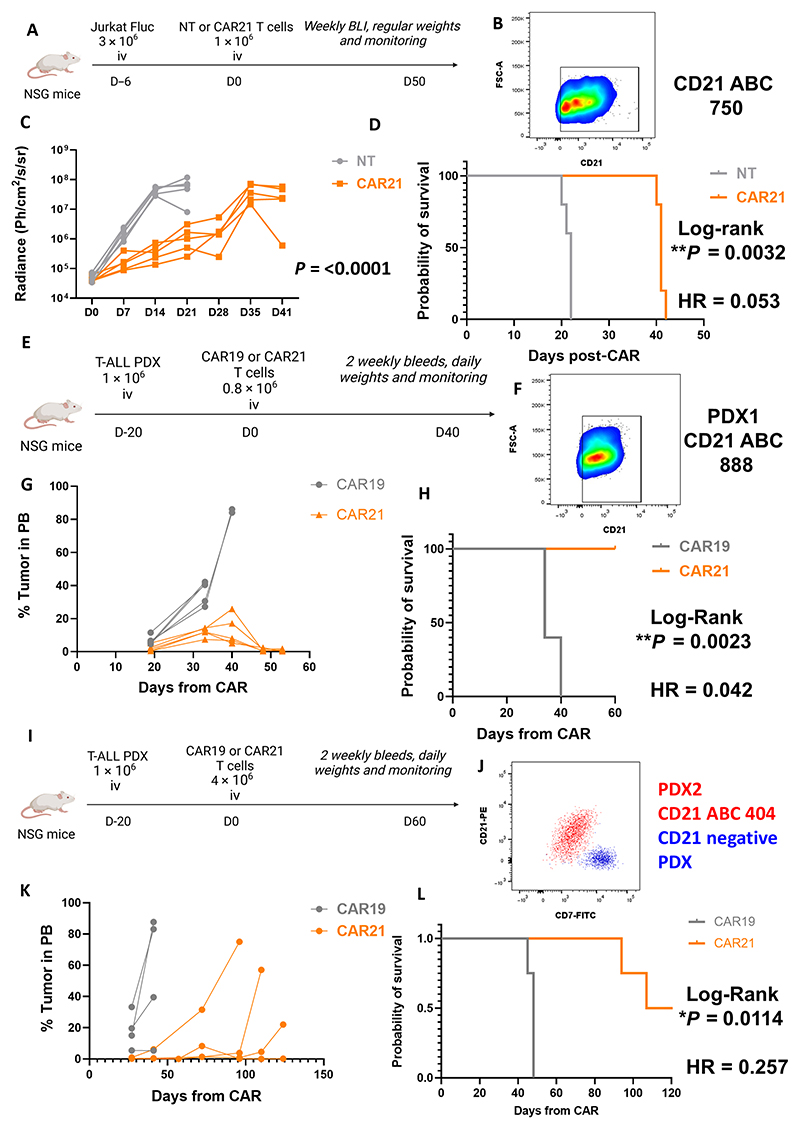
In vivo evaluation of NM2 Fab anti-CD21 CAR-T cells. (**A**) Jurkat murine efficacy model. NSG mice were injected intravenously (iv) with 3 million Jurkat cells followed by 1 million CAR-T 21 or non-transduced T cells on day 6. *n* = 5 per group. (**B**) CD21 expression on Jurkat cells. Gated on isotype control. (**C**) Bioluminescence imaging of NT- and CAR21-treated mice over time. Data were log transformed, and then NT and CAR21 slopes were compared using simple linear regression (*P* = <0.0001). (**D**) Kaplan-Meier survival curve for NT- or CAR21-treated mice, compared by log-rank test. *P* = 0.0034. HR (log rank): 0.05323, 95% confidence interval (CI) of ratio = 0.007492 to 0.3782. (**E**) PDX1 murine efficacy model of T-ALL. One million T-ALL PDX cells were injected intravenously until engraftment followed by 0.8 million CAR-T 21 or CAR-T 19 cells. *n* = 5 per group. (**F**) CD21 expression on PDX1 cells. (**G**) Residual tumor percentage in blood of CAR19-and CAR21-treated mice. (**H**) Kaplan-Meier survival curves for PDX1 mice treated with CAR19 or CAR21 cells. Curves significantly different by log-rank test. *P* = 0.0023, Mantel-Haenszel, HR: 0.04195, 95% CI of ratio 0.005457 to 0.3225. (**I**) PDX2 murine efficacy model. One million T-ALL PDX cells were injected intravenously until engraftment followed by 4 million CAR-T 21 or CAR-T 19 cells. *n* = 4 per group. (**J**) CD21 expression on PDX2 tumor cells. (**K**) Residual tumor cells in blood for CAR19- or CAR21-treated mice post–CAR-T. (**L**) Kaplan-Meier survival curves for PDX2micetreated with CAR19 or CAR21 cells. Comparison by log-rank test. *P* = 0.0114.

NM2 Fab-CAR-T (CAR21) cells were next evaluated in a Jurkat xenograft model of T-ALL. Three million Jurkat cells were injected intravenously into NSG (nonobese diabetic severe combined immunodeficient gamma) mice followed by 1 × 10^6^ CAR21 or NT (nontransduced) T cells intravenously on day 6 (Fig. 6A). Jurkat cells had low but homogeneous expression of CD21 (CD21 ABC: 600 to 750; Fig. 6B). CAR21-treated mice had significantly improved tumor control by bioluminescence imaging (BLI) (*P* = <0.0001 by simple linear regression of transformed radiance; Fig. 6C) and prolonged survival compared with mice that received NT T cells [median 41 versus 22 days, hazard ratio (HR): 0.05323, *P* = 0.0034 by log-rank test; Fig. 6D]. Residual tumor at necroscopy in CD21-CAR recipients showed CD21 down-regulation in spleens (*P* = 0.0013 by unpaired *t* test) but not bone marrow (not significant by unpaired *t* test, *P* = 0.1085) compared with control mice (fig. S15A). There were no persistent detectable T cells at necroscopy.

We next tested CAR21 in two clinically relevant PDX models of T-ALL. In the first PDX (PDX1), 0.8 × 10^6^ CAR21 or CAR19 cells were injected intravenously 20 days after intravenous injection of 1 × 10^6^ PDX cells in NSG mice (Fig. 6E). CD21 was expressed on the cell surface at an ABC of 888 (Fig. 6F). CAR19 recipients experienced rapid disease progression with death by day 40. All CAR21 recipients had no detectable leukemia in blood by day 48 (Fig. 6G). CAR21 mice had a significant survival advantage and were all alive and well at experiment end (*P* = 0.0023 by log-rank test, HR: 0.042; Fig. 6H). Most mice had no detectable CAR-T cells by day 41 (fig. S15B).

The second PDX (PDX2) expressed CD21 at lower antigen density (CD21 ABC: 404). In this model, 1 × 10^6^ T-ALL lymphoblasts were followed by 4 × 10^6^ CAR21 or CAR19 cells on day 20 in NSG mice. Mice were monitored with biweekly bleeding (Fig. 6, I and J). CAR19-treated mice rapidly progressed, and all had died by day 48. CAR21-treated mice had tumor control, with complete disease eradication in the blood by day 57 (Fig. 6K) and improved survival (median survival 48 versus 115 days, HR: 0.257, *P* = 0.0114 by log-rank test; Fig. 6L and fig. S15E). There were late relapses in CAR21 mice in this model leading to death due to increased leukemic burden, with tumor cells detected in spleens from three of four mice at necroscopy. Tumor relapse was associated with loss of detectable CAR-T cells in this immunodeficient model. Tumor control at this stage was achieved in a single mouse with detectable T cells (fig. S15C). Tumor cells at relapse remained CD21 positive (fig. S15D).

### PI3K/mTOR inhibition increases CD21 expression and enhances CAR-T function

In primary T-ALL samples tested, CD21 exhibited a range of antigen density, with some samples potentially falling below the threshold required for effective CAR-T targeting. To address this, we investigated pharmacological strategies to enhance CD21 expression and evaluated their impact on CAR-T cell performance. We focused on the PI3K pathway given that PI3K inhibition of T-ALL cell lines has been demonstrated to activate the NOTCH-MYC pathway and upregulate CD21 (21).

We initially investigated the effect of incubation with PI3K/mTOR/Akt pathway inhibitors [AZD5363 (Akt), GDC0941 (pan PI3K), BEZ235 (pan PI3K+mTOR), and rapamycin (mTOR)] on CD21 expression across six T-ALL cell lines. Rapamycin produced a significant increase in CD21 expression (*P* = 0.0314) with a trend to up-regulation with AZD5363, GDC0941, and BEZ235. Conversely, the γ-secretase inhibitor L685 significantly down-regulated CD21 expression (*P* = 0.0248) (fig. S16A). CD21 modulation persisted up to 48 hours after drug removal (fig. S16B). CD21 modulation did not occur in a CD21-negative cell line, on normal B or T cells (fig. 16C), or in activated T cells (fig. 16D).

**Fig. 7:**
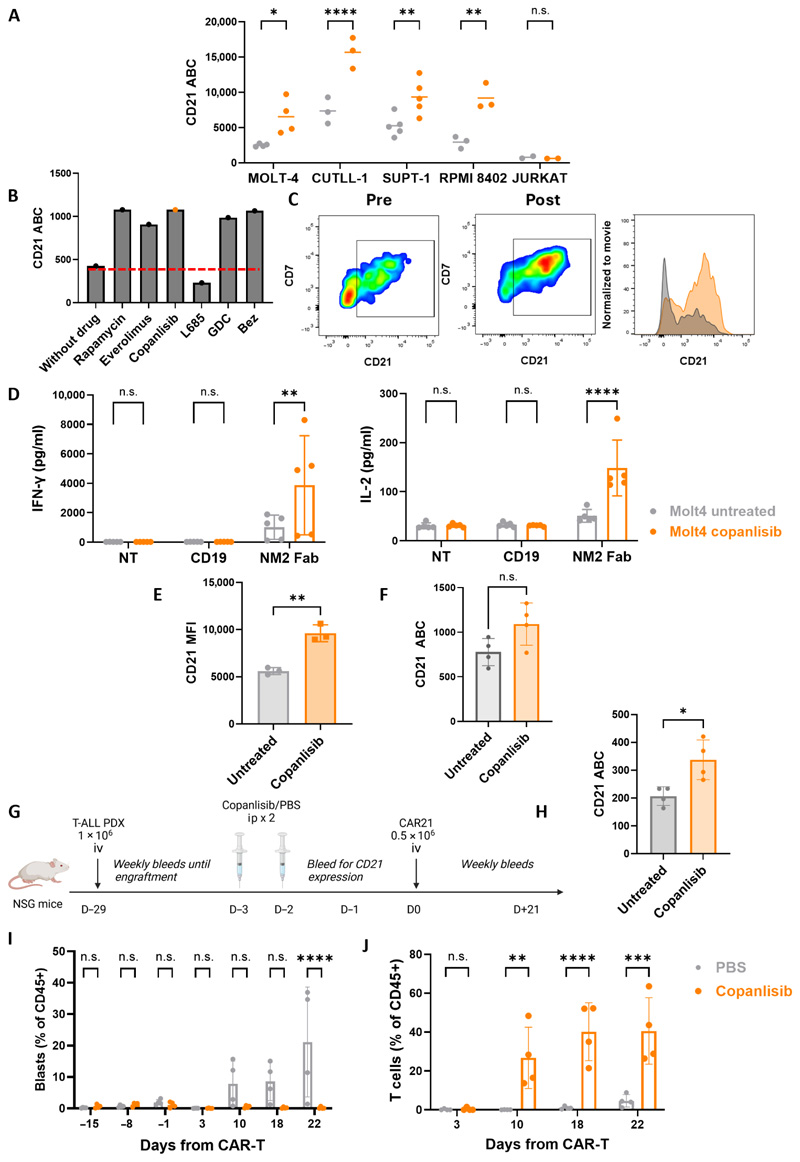
PI3K inhibition increases CD21 expression in T-ALL, enhancing in vitro CD21 CAR-T efficacy. (**A**) CD21 ABC change from baseline in T-ALL cell lines incubated with (orange) and without (gray) copanlisib for 48 hours. MOLT4, *n* = 4; CUTLL-1, *n* = 3; SUPT1, *n* = 5; RPMI 8402, *n* = 3; Jurkat, *n* = 2 experimental repeats. Comparisons made using two-way ANOVA. (**B**) CD21 expression on T-ALL PDX2 lymphoblasts after 48 hours with a range of PI3K inhibitors and L685 in vitro. (**C**) Representative flow cytometry plots and histogram showing expression of CD21 on T-ALL PDX1 lymphoblasts before (left, gray histogram) and after (right, orange histogram) incubation with copanlisib. (**D**) IFN-γ and IL-2 secretion by NM2 Fab CAR21 cells against MOLT4 cells and MOLT4 cells with and without copanlisib. *n* = 5. Comparisons made using two-way ANOVA. Gray: MOLT4-untreated cells; orange: MOLT4 cells pretreated with copanlisib. (**E**) CD21 MFI of bone marrow lymphoblasts in a MOLT4 murine model of T-ALL with or without treatment with two doses of copanlisib [14 mg/kg intra-peritoneally (ip)], *n* = 3 per cohort. (**F**) CD21 ABC on bone marrow lymphoblasts in a T-ALL PDX (PDX1) engrafted in NSG mice with and without intraperitoneal copanlisib. *n* = 4 per cohort. *P* = 0.17 by unpaired *t* test. (**G**) Experimental timeline of a combined copanlisib and CAR21 model in NSG mice engrafted with CD21-low T-ALL PDX3. (**H**) CD21 ABC of T-ALL lymphoblasts in blood with and without two doses of copanlisib (19 mg/kg ip). *n* = 4 per cohort, *P* = 0.0159 by unpaired *t* test. (**I**) % blasts in peripheral blood before and after CAR21 in control-treated (gray) and copanlisib-treated (orange) mice. (**J**) Percentage of T cells in peripheral blood before and after CAR21 in controltreated (gray) and copanlisib-treated (orange) mice. *P = <0.05; **P = <0.01; ***P = <0.001; ****P = <0.0001; n.s., not significant.

We next investigated the clinically approved dual PI3Kαδ inhibitor copanlisib in vitro and saw significant (at least twofold) increases in CD21 expression across four of five T-ALL cell lines tested (MOLT4, *P* = 0.0197; CUTLL-1, *P* = <0.0001; SUPT1, *P* = 0.0069; and RPMI 8402, *P* = 0.0013; Fig. 7A). The impact of copanlisib was specific to CD21 given that, excluding CCR9 on SUPT1, there was no change in CCR9 or CD7 expression across four T-ALL cell lines (fig. S16E). This effect could also be reproduced in a CD21-low PDX T-ALL sample (PDX1) in vitro (Fig. 7, B and C).

We next explored whether PI3K-mediated CD21 up-regulation would improve in vitro CAR21 functionality. In MOLT4 cells, an increase in CD21 after 48-hour preincubation with copanlisib was associated with significantly increased IFN-γ (*P* = 0.0093) and IL-2 (*P* = <0.0001) production by CAR21 T cells (Fig. 7D). This was replicated in cocultures with two further T-ALL cell lines, CUTLL-1 and RPMI 8402 (fig. S16F). Conversely, and as expected given its known inhibitory effect on T cells, when copanlisib was added directly to the coculture rather than removed after preincubation, there was a significant attenuation of IFN-γ (*P* = <0.0001) and IL-2 (*P* = 0.0004) production (fig. S16G).

We lastly investigated the effects of copanlisib on CD21 expression in vivo. CD21 up-regulation after copanlisib treatment was seen in NSG mice engrafted with either MOLT4 cells (Fig. 7E) or a PDX T-ALL sample (PDX2; Fig. 7F). To confirm the hypothesis that copanlisib pretreatment may increase low CD21 expression above a threshold required for CAR-T efficacy, we identified a PDX T-ALL sample with very low CD21 density (PDX3, 207 ABC) and engrafted a further cohort of NSG mice. After 3 weeks, mice were treated with either copanlisib or phosphate-buffered saline (PBS) control. Up-regulation of tumor CD21 expression with copanlisib was confirmed (mean CD21 ABC: 207 PBS versus 338 copanlisib, *P* = 0.0159; Fig. 7H). After a 48-hour washout period (approximately eight half-lives), mice were treated with a low dose (0.5 M) of CAR21 T cells. Tumor control at experiment end was seen only in those mice pretreated with copanlisib (*P* = <0.0001) (Fig. 7I), associated with significant T cell expansion from day 10 onward, *P* = 0.0032 (Fig. 7J) only in these mice. These findings were confirmed in both bone marrow (fig. S16H) and spleens (fig. S16I) of mice at necroscopy.

## Discussion

Patients with r/r T-ALL face a poor prognosis (2, 5, 22). Salvage chemotherapy followed by allogeneic HSCT (allo-HSCT) is the only curative approach but is often unsuccessful and limited to younger patients without comorbidities (23, 24). In contrast, anti-CD19 CAR-T cell therapy for r/r B-ALL can induce durable remission (13). This success has spurred interest in CAR-T cell approaches for r/r T-ALL, where there is an unmet need.

An analogous approach to anti-CD19 CAR-T cell therapy is targeting pan–T cell antigens, with CD7 being the most investigated (8, 9, 25–27). A recent phase 1 study reported 95% bone marrow MRD (minimal residual disease)–negative complete responses (25). However, targeting pan–T cell antigens may cause CAR-T cell fratricide and T cell aplasia. Early anti-CD7 CAR-T data showed expansion of naturally occurring CD7-negative T cells, but overall T cell numbers remained low with opportunistic infections and viral reactivation described, as well as deaths due to monocytopenia (8, 28, 29). Phase 2 data with longer follow-up revealed that most patients relapsed without consolidative transplant, and 30 to 50% experienced CD7 antigen loss (8, 28).

An alternative approach is targeting selective antigens expressed by T-ALL blasts but not by normal T cells. CD1a is one such selective target, although it has only been identified as expressed by cortical T-ALL which tends to have a good prognosis (11). Anti-TRBC1/2 CAR-T cells are more relevant to mature T cell malignancies given that only one-third of patients with T-ALL express surface TCR (T cell receptor) (10). We recently proposed CCR9 as a CAR target for T-ALL; however, it is not expressed in all T-ALL cases, with 65% positivity at diagnosis (12). Given the lack of a ubiquitously expressed selective T-ALL target antigen and the likelihood of target antigen modulation, identifying additional T-ALL targets is necessary.

Here, we explored CD21 as a leukemia-selective CAR target in T-ALL. CD21 is also found in mature B cell malignancies such as CLL (chronic lymphocytic leukemia), DLBCL (diffuse large B cell lymphoma), and follicular NHL (non-Hodgkin’s lymphoma) (30–32). Reports of CD21 expression in T cell malignancies are limited, although CD21 positivity of T-ALL cell lines and T-ALL patient samples has been previously reported (33, 34). Recent studies by the Tokyo Children’s Cancer Study Group described CD21 expression in T-ALL, with the highest expression in cortical T-ALL (35). Our study confirms uniform CD21 expression in approximately half of T-ALL patient samples but on <10% of normal T cells.

We compared the expression of CD21 with other T-ALL selective targets currently undergoing clinical exploration, CCR9 and CD1a. Expression of CD21 with either antigen was only partially overlapping. A cotargeting approach with CD21 CAR and either CCR9 or CD1a CAR would both increase the proportion of patients who could receive antigen-restricted CAR-T treatment and potentially reduce the risk of antigen-negative escape.

CD21 is expressed on normal B cells and FDCs, so B cell aplasia is an expected limitation of CD21-directed CAR-T therapy, but it is generally well tolerated (36). Depletion of FDCs could impair responses to infections. In mature αβ T cells, CD21 was expressed at very low density on only <10%, with the highest expression in naïve subsets, as described in the literature (37). Accordingly, anti-CD21 CAR-T cells were not affected by fratricide, and T cell aplasia is an unlikely consequence of therapy. The functional consequences of CD21+ T cell depletion are unknown, but *CR2* KO mice (which lack both CD21 and CD35) exhibit impaired IgM and IgG responses without a T cell deficit. Previous antibody-based CD21 therapies for B cell malignancies resulted in minimal toxicity (38, 39).

Of note, CD21 was expressed on a small proportion (mean 15%) of γδ T cells, suggesting that anti-CD21 CAR-T could lead to partial depletion of these cells. There are no data on the role of CD21 in γδ T cells or whether CD21+ γδ T cells represent a unique subtype. Furthermore, pan–γδ T cell depletion presents largely unknown clinical consequences. Theoretically, there may be a compromise of early-stage immune responses against intracellular pathogens and tumors, although murine models suggest that the phenotype may be relatively mild. Detailed exploration of this issue will require clinical testing.

CD21 is expressed at relatively low density in T-ALL (~1300 ABC) compared with CD19 in B-ALL (>10,000 ABC) (40) but similar to other successful CAR targets like B cell maturation antigen (~1500 ABC) (41). Although anti-CD21 CAR-T cells were effective at low CD21 density, there is likely a correlation between antigen density and CAR-T potency (40). We found that inhibiting the PI3K/mTOR pathway up-regulated CD21 in T-ALL, enhancing CAR-T cell efficacy both in vitro and in vivo. Copanlisib pretreatment enabled CAR-T control of a PDX sample with a CD21 ABC of only 207, in which CAR-T cells alone were not effective. Previously, we showed that PI3K inhibition led to increased activation of NOTCH target genes, increased *MYC* RNA expression, and increased surface CD21 expression in T-ALL cells (21). This was likely mediated by glycogen synthase kinase-3 (GSK-3) (42–44). However, PI3K inhibitors can impair T cell function (45), so careful clinical strategies will be needed to balance these effects. These could include sequential targeting/intermittent use of PI3K inhibition to up-regulate CD21 density while minimizing inhibitory effects on T cells.

A further challenge for CD21 as a CAR-T target is its long, flexible, highly glycosylated structure (46, 47). At 146 kDa, it is larger than well-studied CAR-T targets like CD19 and even CD22 (48). Our initial scFv-based anti-CD21 CARs targeting membrane-distal epitopes were not functional, mirroring findings with CARs targeting membrane-distal CD22 epitopes (49). We therefore tested CARs with scFvs recognizing CD21 membrane-proximal epitopes. These resulted in some anti-CD21 activity but remained considerably less potent than anti-CD19 CAR-T cells.

We next combined the targeting of membrane-proximal epitopes with a Fab-CAR architecture, hypothesizing that the increased stability of the Fab format would enhance potency. CD21 Fab-CARs demonstrated improved efficacy, prompting further investigation into the underlying mechanisms. Initially, we considered whether increased stability might lead to higher expression of Fab-CARs; however, Fab-CARs were found to be expressed at lower surface densities compared with scFv-CARs, likely because of the bicistronic expression system required. We then questioned whether this reduced expression could paradoxically enhance function. To test this, we generated an scFv-CAR with an upstream IRES element, which not only reduced surface expression and resulted in enhanced cytotoxicity but also diminished cytokine secretion. This suggested that reduced expression alone does not account for the superior performance of anti-CD21 Fab-CARs.

We observed that multiple CD21 scFv-CARs exhibited degrees of tonic signaling that were reduced in Fab-CARs, which contributed to enhanced antitumor efficacy (48, 49). This reduction was likely due to the increased thermal stability of Fab binders (50) and their reduced tendency for clustering, which can occur in scFvs because of VH-VL domain instability and unfolding (51–53).

In addition, we demonstrated that the use of a CD28 TMD instead of a CD8 TMD enhanced CAR potency, as previously reported, although this did not mitigate tonic signaling (40). CD21 Fab-CARs exhibited equivalent binding affinity but lower cell-to-cell avidity compared with their scFv counterparts. Although higher avidity is often considered beneficial for CAR-T efficacy, this is not consistently supported across all datasets (54). Our findings high-light the complexities of CAR design, emphasizing that, although some principles for optimal CAR-T constructs are established, empirical testing remains essential, particularly when targeting challenging antigens.

A final anti-CD21 Fab-CAR was effective against multiple T-ALL cell lines and patient samples in vitro and in clinically relevant in vivo models. In a Jurkat xenograft model, CAR21-treated mice had statistically improved survival over control mice. CD21 down-regulation in the absence of CAR-T cells was seen in the spleens of mice at relapse, suggesting possible antigen-negative escape. Encouragingly, this was not seen in more clinically relevant PDX models. Antigen-negative escape is a potential risk of all single antigen–targeting CAR-T cells and has emerged in recent clinical trials of CD7-targeting CAR-T cells. Ultimately, clinical testing is required to fully evaluate this risk in patients.

Our study has some limitations. Most patient samples analyzed were from pediatric patients, and it is possible that the expression of CD21 may differ in an adult population. In addition, because of the rarity of the disease, the number of r/r patient samples that were available to us was relatively limited. Lastly, although our findings demonstrate the efficacy of anti-CD21 CAR-T cells in relevant preclinical models of T-ALL, well-designed clinical trials will be necessary to confirm their potential in patients.

Early CAR-T data in T-ALL show promise, but current clinical-stage approaches that target pan–T cell antigens require allo-HSCT to consolidate responses and mitigate toxicity. Use of antigens selectively expressed by T-ALL blasts may avoid the limitations of these approaches. CD21 adds to such targets because it is expressed by approximately half of T-ALL cases but only on a small population of normal T cells. CD21 is a large and bulky antigen. However, anti-CD21 Fab-CAR-T cells showed potent anti-CD21 activity, and pharmacological up-regulation of CD21 further enhanced efficacy. Anti-CD21 CAR-T cell targeting may open avenues in the treatment of T-ALL while avoiding fratricide and T cell aplasia.

## Materials and Methods

### Study design

This study was designed to validate expression of CD21 in T-ALL and normal tissues using cell lines, patient samples, and healthy donors. The study additionally involved identification of CD21-directed antibody fragments followed by preclinical testing of anti-CD21 CAR-T cells using in vitro assays (cell lines, patient samples, and PDX models) and in vivo models of cell lines and PDX models. Except where stated, all in vitro experiments were carried out on at least two separate occasions and had three technical replicates for each condition. *P* values were determined using methods described in the figure legends. For all in vivo experiments, a minimum of four mice were used per arm in each animal model. Mice were randomly assigned to experimental groups. Investigators were blinded to experimental circumstances during assessment and data analysis. All mice were cared for in an unbiased fashion by animal technicians. Given the discovery research nature of the study, no predefined power calculations for sample size were used.

### Cell lines and maintenance

The human embryonic kidney (HEK) 293T cell line was cultured in Iscove’s modified Dulbecco’s medium (IMDM; Lonza) with 10% fetal bovine serum (FBS) (HyClone, GE) and 2 mM GlutaMAX (Invitrogen). All others were cultured in RPMI (Lonza) with 10% FBS and 2 mM GlutaMAX. All underwent routine mycoplasma testing and were obtained from the Deutsche Sammlung von Mikroorganismen und Zellkulturen (DSMZ).

### Samples and flow cytometry

The UCL Research Ethics Committee granted ethical approval. Whole blood was obtained from consenting healthy donors or leukocyte cones from the NHS Blood and Transplant. PBMCs were isolated using Ficoll-Paque (GE Healthcare) and SepMate tubes (STEMCELL technologies) following the manufacturer’s protocol.

T-ALL peripheral blood and bone marrow samples were obtained from the UCL/UCLH Biobank, Great Ormond Street Biobank, Blood Cancer UK Leukaemia CellBank, and the Biobank of Ospedale Papa Giovanni XXIII, Bergamo, Italy. Flow cytometry was performed on a BD LSR Fortessa instrument (BD Biosciences), a CytoFLEX (Beckman Coulter), or NovoCyte (Agilent) 96-well flow cytometer. CD21 antigen density (ABC) was determined using Quantibrite PE beads (BD) as per the manufacturer’s instructions.

### CD21-scFv generation

Genetic rat vaccination for CD21 was performed by Aldevron. Three rats were DNA vaccinated with a pVAC (DNA vaccine) plasmid engineered to express the full ectodomain and TMD of CD21 with a truncated endodomain (UniProt P20023-1 amino acid sequence 1 to 1014; fig. S17A). In a second vaccination, a further three rats were vaccinated with a pVAC plasmid expressing only SCRs 11 to 15 of the CD21 ectodomain with a truncated endodomain (UniProt P20023-1 amino acid sequence 660 to 1014; fig. S17B). Animals were culled once seroconversion was confirmed. Immune phage libraries were created using an in-house protocol. Anti-CD21 scFvs were identified through biopanning and then screening of these immune phage display libraries both manually and using PacBio next-generation sequencing (Genewiz), as previously described (20). Briefly, phage libraries were incubated with CD21-coated streptavidin beads for two or three rounds to enrich the libraries for anti-CD21 antibodies. Once bulk library enrichment for CD21 was confirmed, the supernatant from individual bacterial colonies was assessed for binding to CD21 by flow cytometry. DNA was extracted from colonies showing the greatest binding to CD21 and sequenced using Sanger sequencing. In addition, DNA from the second round of biopanning was amplified using pHEN1 phagemid vector–specific primers. Amplicon DNA was then sequenced using Pacific Biosciences sequencing technology by Genewiz.

### Surface plasmon resonance

Kinetic analysis was performed on a Biacore T200 (GE Healthcare) using recombinant human CD21 protein covalently coupled to a CM5 sensor chip according to the manufacturer’s recommendations. Serial dilutions of scFvs with a murine IgG2a Fc were captured and injected over the flow cells at a rate of 30 μl/min, and equilibrium binding analysis was performed. Kinetic rate constants were obtained by curve fitting according to a Langmuir 1:1 binding model. Data analysis was performed using Biacore Insight Evaluation software v3.0 (GE Healthcare).

### Differential scanning fluorimetry

A Prometheus NT.48 (NanoTemper) was used. The emission of fluorescence radiation was measured at wavelengths 330 and 350 nm across a temperature range from 20° to 95°C at a rate of increase of 1°C min^−1^ and used to determine the protein melting temperatures.

### Avidity measurements

MOLT4 cells were seeded on poly-l-lysine–coated z-Movi microfluidic chips at a density of 160 × 10^6^ cells/ml. Cells were incubated for 0.5 hour in serum-free medium followed by a 2-hour incubation in complete medium. Effector cells were stained with CellTrace far red (Thermo Fisher Scientific, C34564) at 1:000 dilution. Labeled effector cells were introduced on to the chip at a density of 10 × 10^6^ and incubated for 10 min before acoustic force application using a z-Movi cell avidity analyzer. Detachment of effector cells was analyzed using Oceon 1.4 software.

### Generation of CAR-T cells

CAR constructs were expressed in the SFG vector unless otherwise stated and the retroviral supernatant generated through transient transfection of HEK293T cells. Donor PBMCs were transduced using a standardized protocol as previously described (12).

### Generation of engineered cell lines

Raji and SUPT1 CD21 KO cell lines created using CRISPR-Cas9 engineering were donated by Autolus Ltd. Retroviral vectors containing CD21 SCRs 1 to 5, 6 to 10, and 11 to 15 and a blue fluorescent protein (BFP) marker were used to transduce SUPT1 and Raji CD21 KO cell lines. Cells were bulk sorted for CD21+ BFP+ populations and then single-cell cloned.

### In vitro cytotoxicity and cytokine release assays

Target cells were stained with CellTrace violet (CTV) (Thermo Fisher Scientific), plated at 25,000 to 50,000 per well with three technical repeats. CAR-T cell–to–target cell ratios of 1:1, 1:2, 1:4, 1:8, and 1:16 were used. Controls were NT cells, a nonbinding aCD21 CAR (Ph2), and aCD19 FMC63 CAR. At 48 hours, enzyme-linked immunosorbent (ELISA) assay (BioLegend) for IFN-γ and IL-2 was performed on the cell supernatant. Seventy-two hours of coculture of primary T-ALL cells was performed using allogeneic donor T cells. Residual lymphoblasts were identified using human CD45, murine CD45, CD3, and CTV staining.

### In vitro proliferation assays

CAR-T cells labeled with CTV were cocultured with irradiated (30Gy) target cells in a 1:2 E:T (effector-to-target) ratio for 7 days. CTV median fluorescence intensity (MFI) on days 0 and 7 determined CTV dilution and fold expansion from baseline. CAR-T cell counts were established by staining with aCD34 antibody for the RQR8 marker gene.

### Jurkat NFAT reporter cell line assays

Jurkat cells were engineered to express enhanced green fluorescent protein (GFP) under the control of the NFAT promoter. The *CR2* gene was deleted from cells by CRISPR-Cas9 and single-cell cloned to produce a Jurkat_NFAT-GFP_CD21KO reporter cell line. Anti-CR2 guide RNA was designed using CRISPOR (CTC-TAGGACGATTTCCCAAT). This line was transduced with CAR constructs, and GFP surface expression in transduced cells was assayed on day 7.

### Tonic signaling assays in PBMCs

Healthy donor PBMCs were transduced with CAR constructs and stained for CTV as previously described. A total of 100,000 transduced cells per well were plated without targets or exogenous cytokines. Readouts of CTV MFI, cytokine secretion, and exhaustion/differentiation were performed on days 0, 7, 9, 12, and 14.

### In vivo models of T-ALL

Work was performed under a UK Home Office–approved project license (PP8379762) and was approved by the UCL Biological Services Ethical Review Committee. PDX1 was derived from a 10-year-old boy with biallelic CDKN2A-deleted T-ALL. PDX2 was derived from a 1-year-old girl with ATM-deleted T-ALL. PDX3 was from a 14-year-old boy with a STIL deletion. All were developed at the Institute of Child Health by O. Williams or in house.

Female and male NSG mice aged >8 weeks (Charles River) were used, with groups matched for sex. Mice were culled over behavioral concerns, >15% weight loss, or at prespecified time points. Cell suspensions were injected by tail vein. Fifty microliters of a tail vein bleed was taken at set time points. Tumor cells were identified as human CD45+ CD3-negative. BLI was performed using IVIS (PerkinElmer). General anesthesia was induced using inhaled isoflurane. After induction, intraperitoneal injection of luciferin (200 μl) was undertaken.

### Pharmacological manipulation of CD21 antigen density

The drug concentrations used were L685 (1 μM), GDC0941 (1 μM), BEZ235 (1 μM), AZD5363 (5 μM), rapamycin (20 nM), and copanlisib (100 nM). Target cells at 5 × 10^5^/ml were incubated with drugs in 24-well or 6-well T cell–coated plates for 24, 48, or 72 hours. T lymphoblasts were maintained in culture using the HS5 stromal cell line, donated by K. Yong. Blasts were identified as CD7+ BFP-negative. Drugs were washed off three times using sterile PBS. In vivo, copanlisib (14 or 19 mg/kg) or DMSO (dimethyl sulfoxide)/PBS control was injected intraperitoneally on 2 consecutive days, and CD21 expression was assessed at 24 hours posttreatment.

### Statistical analysis

Statistical analysis was performed using GraphPad Prism v9 for Windows (GraphPad Software). Student’s *t* test or two-way analysis of variance (ANOVA) was used for samples with normally distributed variables as indicated in the text. Survival curves were generated using the Kaplan-Meier method with HRs calculated by the log-rank method. *P* < 0.05 showed statistical significance. Data are summarized as means ± SD unless otherwise specified.

## Supplementary Material

MDAR reproducability checklist

Supplementary methods anbd figures

## Data Availability

All data associated with this study are present in the paper or the Supplementary Materials. All plasmids used in the study including CAR-T constructs and CD21-truncation expression vectors are available from the corresponding author, N.M., upon request and completion of a material transfer agreement.
