# Supplementary material for: Preclinical development of anti-CD21 chimeric antigen receptor T cells to treat T cell acute lymphoblastic leukemia
Source: Sci Transl Med. Author manuscript; Available in PMC 2025 Apr 30. (PMC7617635; doi:10.1126/scitranslmed.adr1476)
Supplement: MDAR reproducability checklist [file EMS204776-supplement-MDAR_reproducability_checklist.pdf]

Materials Design Analysis Reporting (MDAR)
Checklist for Authors

The MDAR framework establishes a minimum set of requirements in transparent reporting applicable to studies in the life sciences (see Statement of Task: doi:10.31222/osf.io/9sm4x.). The MDAR checklist is a tool for authors, editors, and others seeking to adopt the MDAR framework for transparent reporting in manuscripts and other outputs. Please refer to the MDAR Elaboration Document for additional context for the MDAR framework.

For all that apply, please note where in the manuscript the required information is provided.

Materials:

Newly created materials	indicate where provided: page no/section/legend)	n/a
The manuscript includes a dedicated "materials availability statement" providing transparent disclosure about availability of newly created materials including details on how materials can be accessed and describing any restrictions on access.	A data availability statement is available on line 840 of the manuscript.	

Antibodies	indicate where provided: page no/section/legend)	n/a
For commercial reagents, provide supplier name, catalogue number and RRID, if available.	A list of all antibodies used with their suppliers and catalog numbers can be found in supplementary methods.	

DNA and RNA sequences	indicate where provided: page no/section/legend)	n/a
**Short novel DNA or RNA including primers, probes:** Sequences should be included or deposited in a public repository.	Guide RNA targeting specific sequences in CD21 was ordered from listed supplier (IDT) and sequence used can be found on line 566 in materials and methods.	

Cell materials	indicate where provided: page no/section/legend	n/a
**Cell lines:** Provide species information, strain. Provide accession number in repository **OR** supplier name, catalog number, clone number, **OR** RRID.	Cell line supplier and culture conditions are provided in materials and methods (lines 484-488)	
**Primary cultures:** Provide species, strain, sex of origin, genetic modification status.	Details of primary human T cell extraction technique and culture conditions are details in materials and methods on lines 491.	

Experimental animals	indicate where provided: page no/section/legend)	n/a
**Laboratory animals or Model organisms:** Provide species, strain, sex, age, genetic modification status. Provide accession number in repository **OR** supplier name, catalog number, clone number, **OR** RRID.	Details of laboratory animals used including supplier, species, strain, sex, age and genetic modification status are provided in materials and methods lines 573-586. Jackson Laboratory NSG mice Strain #:005557, IMSR_JAX:005557 were purchased from Charles River.	
**Animal observed in or captured from the field:** Provide species, sex, and age where possible.		x

Plants and microbes	indicate where provided: page no/section/legend)	n/a
**Plants:** provide species and strain, ecotype and cultivar where relevant, unique accession number if available, and source (including location for collected wild specimens).		x
**Microbes:** provide species and strain, unique accession number if available, and source.		x

Human research participants	indicate where provided: page no/section/legend) or state if these demographics were not collected	n/a
If collected and within the bounds of privacy constraints report on age, sex and gender or ethnicity for all study participants.	All patient samples were anonymized at the point of collection and so details on age, sex, gender or ethnicity are not available.	

Design:

Study protocol	indicate where provided: page no/section/legend)	n/a
If study protocol has been pre-registered, provide DOI. For clinical trials, provide the trial registration number **OR** cite DOI.		x

Laboratory protocol	indicate where provided: page no/section/legend)	n/a
Provide DOI **OR** other citation details if detailed step-by-step protocols are available.		x

Experimental study design (statistics details)		
**For in vivo studies:** State whether and how the following have been done	**indicate where provided: page no/section/legend. If it could have been done, but was not, write not done**	**n/a**
Sample size determination	As this was discovery research, study size calculations were not done.	
Randomisation	For all animal work, animals were randomly assigned to experimental arms.	
Blinding	For all animal work, investigators were blinded to the experimental arms during the experiment and analysis and unblinding was only done at experiment end. Details of all the above are provided in study design section on line 471.	
Inclusion/exclusion criteria	Not done	

Sample definition and in-laboratory replication	indicate where provided: page no/section/legend	n/a
State number of times the experiment was replicated in laboratory.	For each experiment where possible at least three replicates was performed where samples allowed unless otherwise specified in the manuscript. For some experiments with patient samples, only one replicate was performed due to the limited number of cells available.	
Define whether data describe technical or biological replicates.	For each experiment, three technical replicates were performed where feasible. For experiments involving healthy donors, at least three biological replicates were performed where possible to allow for statistical comparison. For experiments involving patient samples, due to the small number of cells available, in some experiments only one biological replicate was performed. Details of number of biological replicates for each experiment are provided in the relevant figure legends.	

Ethics	indicate where provided: page no/section/legend	n/a
**Studies involving human participants:** State details of authority granting ethics approval (IRB or equivalent committee(s), provide reference number for approval.	Ethical approval for use of human samples is detailed on line 490. Ethical approval for the use of patient and healthy donor samples was obtained from the UCL Research Ethics Committee (REC) (Project ID 12807/001 and 12807/004).	
**Studies involving experimental animals:** State details of authority granting ethics approval (IRB or equivalent committee(s), provide reference number for approval.	Animal work was performed under a UK Home Office-approved project license (PP8379762) and was approved by the UCL biological services ethical review committee as detailed on lines 574-575.	
**Studies involving specimen and field samples:** State if relevant permits obtained, provide details of authority approving study; if none were required, explain why.		x

Dual Use Research of Concern (DURC)	indicate where provided: page no/section/legend	n/a
If study is subject to dual use research of concern regulations, state the authority granting approval and reference number for the regulatory approval.		x

Analysis:

Attrition	indicate where provided: page no/section/legend	n/a
Describe whether exclusion criteria were preestablished. Report if sample or data points were omitted from analysis. If yes report if this was due to attrition or intentional exclusion and provide justification.	Exclusion criteria were not pre-established.	

Statistics	indicate where provided: page no/section/legend	n/a
Describe statistical tests used and justify choice of tests.	Statistical analysis was performed using GraphPad Prism v9 for Windows (GraphPad Software). Student's t test or 2-way ANOVA were used for samples with normally-distributed variables as indicated in the text. Survival curves were generated using the Kaplan-Meier method with hazard ratios calculated by the log-rank method. $p < 0.05$ showed statistical significance. Data is summarized as mean +/- standard deviation unless otherwise specified. This information can be found in materials and methods on lines 596-600.	

Data availability	indicate where provided: page no/section/legend	n/a
For newly created and reused datasets, the manuscript includes a data availability statement that provides details for access or notes restrictions on access.		x
If newly created datasets are publicly available, provide accession number in repository **OR** DOI **OR** URL and licensing details where available.		x
If reused data is publicly available provide accession number in repository **OR** DOI **OR** URL, **OR** citation.		x

Code availability	indicate where provided: page no/section/legend	n/a
For all newly generated custom computer code/software/mathematical algorithm or re-used code essential for replicating the main findings of the study, the manuscript includes a data availability statement that provides details for access or notes restrictions.		X
If newly generated code is publicly available, provide accession number in repository, **OR** DOI **OR** URL and licensing details where available. State any restrictions on code availability or accessibility.		x
If reused code is publicly available provide accession number in repository **OR** DOI **OR** URL, **OR** citation.		x

Reporting

MDAR framework recommends adoption of discipline-specific guidelines, established and endorsed through community initiatives. Journals have their own policy about requiring specific guidelines and recommendations to complement MDAR.

Adherence to community standards	indicate where provided: page no/section/legend	n/a
State if relevant guidelines (e.g., ICMJE, MIBBI, ARRIVE) have been followed, and whether a checklist (e.g., CONSORT, PRISMA, ARRIVE) is provided with the manuscript.		x
