# Supplementary material for: Preclinical development of anti-CD21 chimeric antigen receptor T cells to treat T cell acute lymphoblastic leukemia
Source: Sci Transl Med. Author manuscript; Available in PMC 2025 Apr 30. (PMC7617635; doi:10.1126/scitranslmed.adr1476)
Supplement: Supplementary methods anbd figures [file EMS204776-supplement-Supplementary_methods_anbd_figures.pdf]

Science Translational Medicine
AAAS

Supplementary Materials for

Preclinical development of anti-CD21 chimeric antigen receptor T cells to treat T cell acute lymphoblastic leukemia

Nicola Maciocia *et al.*

Corresponding author: Nicola Maciocia, n.maciocia@ucl.ac.uk

Sci. Transl. Med. **17**, eadr1476 (2025)
DOI: 10.1126/scitranslmed.adr1476

The PDF file includes:

Methods
Figs. S1 to S17

Other Supplementary Material for this manuscript includes the following:

Data file S1
MDAR Reproducibility Checklist

SUPPLEMENTARY METHODS

Flow cytometry panels

PBMCs were identified using optimised antibody staining panel: CD3 PB (Biolegend), CD4 BV650 (Biolegend), CD8 PE-Cy7 (Biolegend), CD11b Viogreen (Miltenyi), CD14 BV785 (Biolegend), CD19 PE e610 (eBioscience), CD21 PE (Biolegend), CD45 FITC (Biolegend), CD56 APC (Biolegend), Gamma-Delta PerCP-Cy5.5 (Biolegend), Viability APC-780 (eBioscience).

T cells subsets and exhausted T cells were identified using the following optimized staining panels. Panel 1: Viability APCe780 (eBioscience), CD3 FITC (Biolegend), CD4 BV650 (Biolegend), CD8 AF700 (Biolegend), CD19 PEe610 (eBioscience), CD45 RA APC (Biolegend), CCR7 PerCPCy5.5 (Biolegend), CD95 PE-cy7 (eBioscience). Panel 2: Viability APCe780 (eBioscience), CD3 FITC (Biolegend), CD4 BV650 (Biolegend), CD8 AF700 (Biolegend), CD19 PEe610 (eBioscience), TIM3 APC (Biolegend), PD-1 BV421 (Biolegend), LAG3 PECy7 (Biolegend), CD57 BV786 (Biolegend), Gamma/delta TCR PerCPCy5.5 (Biolegend). Panel 3: Viability APCe780 (eBioscience), CD3 FITC (Biolegend), CD4 BV650 (Biolegend), CD8 AF700 (Biolegend), CD19 PEe610 (eBioscience), CD25 BV786 (BD biosciences), FOXP3 BV421 (Biolegend).

T-ALL patient samples were characterised using the following panels - Panel 1: CD1a PerCPCy5.5 (Biolegend), CD3 PB (Biolegend), CD4 BV650 (Biolegend), CD5 BV510 (Biolegend), CD7 APC (Biolegend), CD8 PECy7 (Biolegend), CD19 PE e610 (eBioscience), CD45 FITC (Biolegend), Viability APC-780 (eBioscience), CD21 PE Bu32 (Biolegend), CD21 IC MOPC21 (Biolegend). Panel 2: CD2 PerCPCy5.5 (Biolegend) HLA-DR PB (Biolegend), CD33 BV650 (Biolegend), CD117 BV510 (Biolegend), CD7 APC (Biolegend), CD34 PECy7 (Biolegend), CD19 PEe610 (eBioscience), CD45 FITC (Biolegend), Viability

APC-780 (eBioscience), CCR9 PE (Biolegend), CCR9 IC MOPC173 (Biolegend). T cell leukemic blasts and T lymphocytes were identified using standard CD45 vs SSC gating followed by exclusion of CD19/CD21 positive B lymphocytes. CD21 gating was performed using an isotype control antibody MOPC-21 (Biolegend). An example gating strategy for primary T-ALL samples is shown in Figure S3B.

CAR-T cells were stained for exhaustion and differentiation markers. Panel 1: Viability APC-780 (eBioscience), hCD34 RQR8 PE (R&D systems), CD28/CD27 PECy7 (Biolegend), CD4 BV650 (Biolegend), CD8 AF700 (Biolegend), CD45 RA APC (Biolegend), CCR7 PerCPCy5.5 (Biolegend). Panel 2: Viability APC-780 (eBioscience), CD34 RQR8 PE (R&D systems), CD4 BV650 (Biolegend), CD8 AF700 (Biolegend), TIM-3 APC (Biolegend), PD1 PerCPCy5.5 (Biolegend), LAG3 PECy7 (Biolegend).

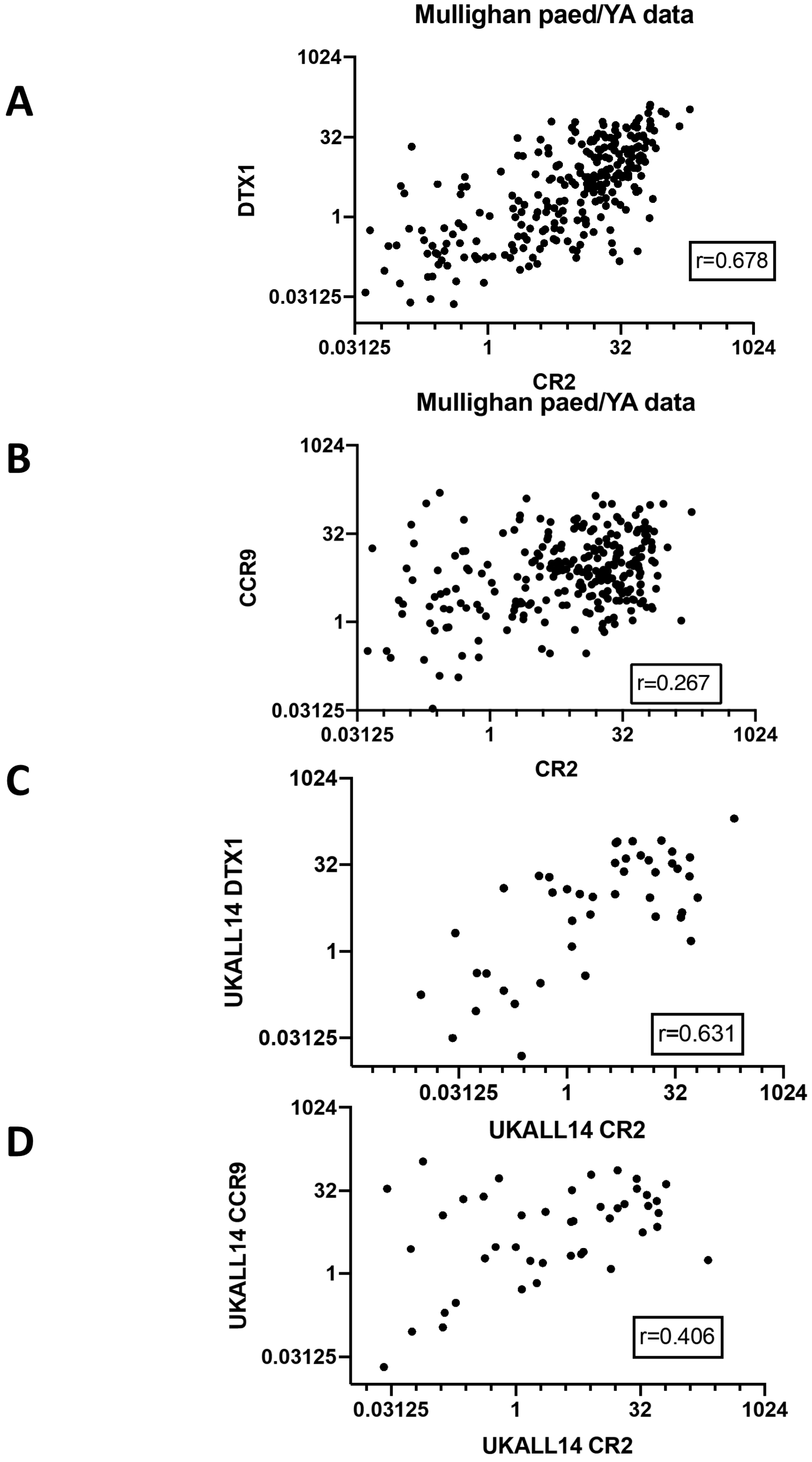
A
Mulligan paed/YA data
DTX1
CR2
r=0.678
B
Mullighan paed/YA data
CCR9
CR2
r=0.267
C
UKALL14 DTX1
UKALL14 CR2
r=0.631
D
UKALL14 CCR9
UKALL14 CR2
r=0.406
1024
32
1
0.03125

Figure S1: Comparison of *CR2* (CD21) RNA expression with *DTX1* (deltex1) and *CCR9*. Data from both pediatric (**A-B**) and adult (**C-D**) T-ALL datasets (Liu et al 2017, Mansour et al 2019)

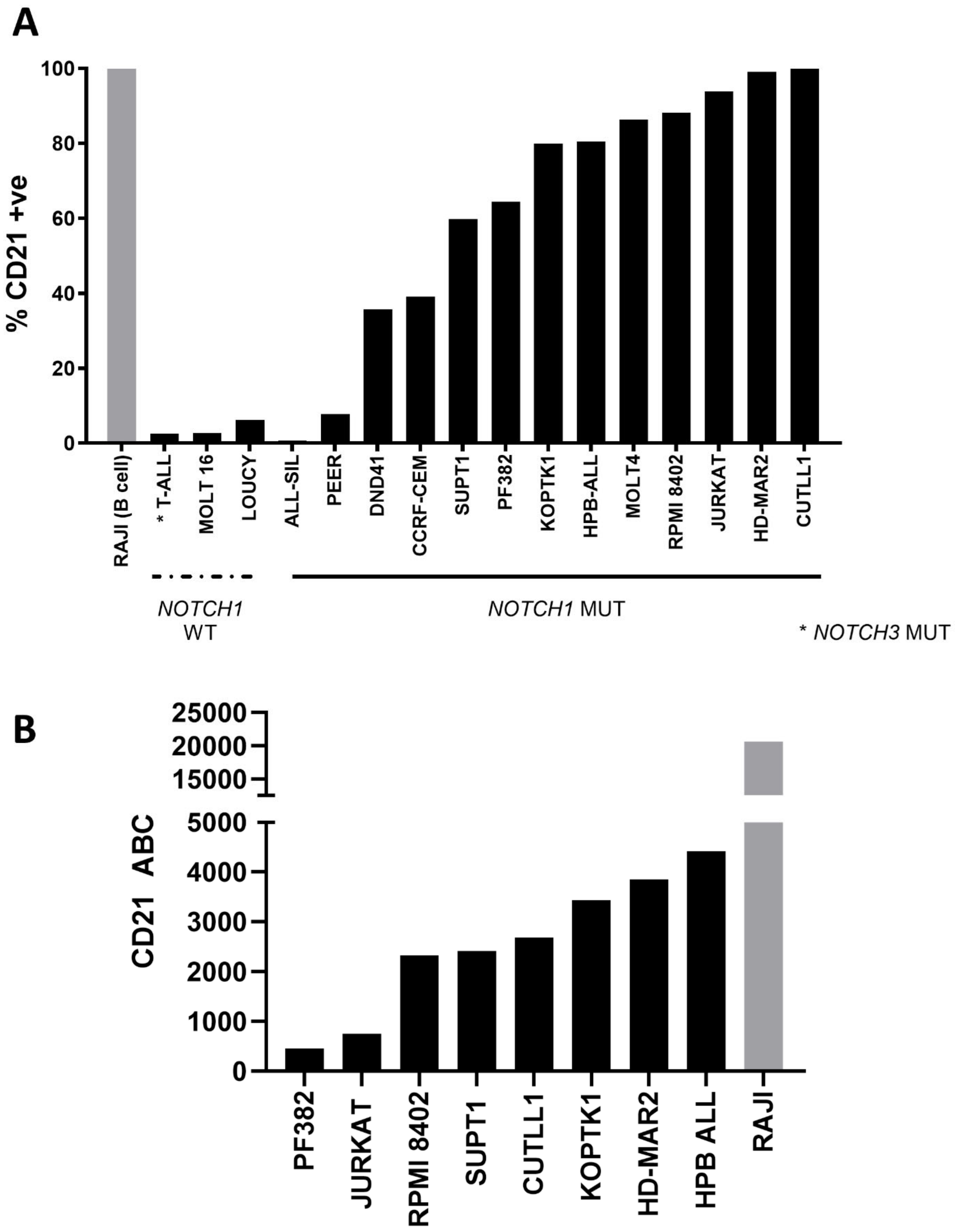

Figure S2: (**A**) Percentage expression of CD21 by flow cytometry across 16 T-ALL cell lines and Raji B-NHL cell line, listed according to *NOTCH1* mutation status (**B**) CD21 antigen density of T-ALL cell lines relative to Raji cell line.

Diagnostic

A

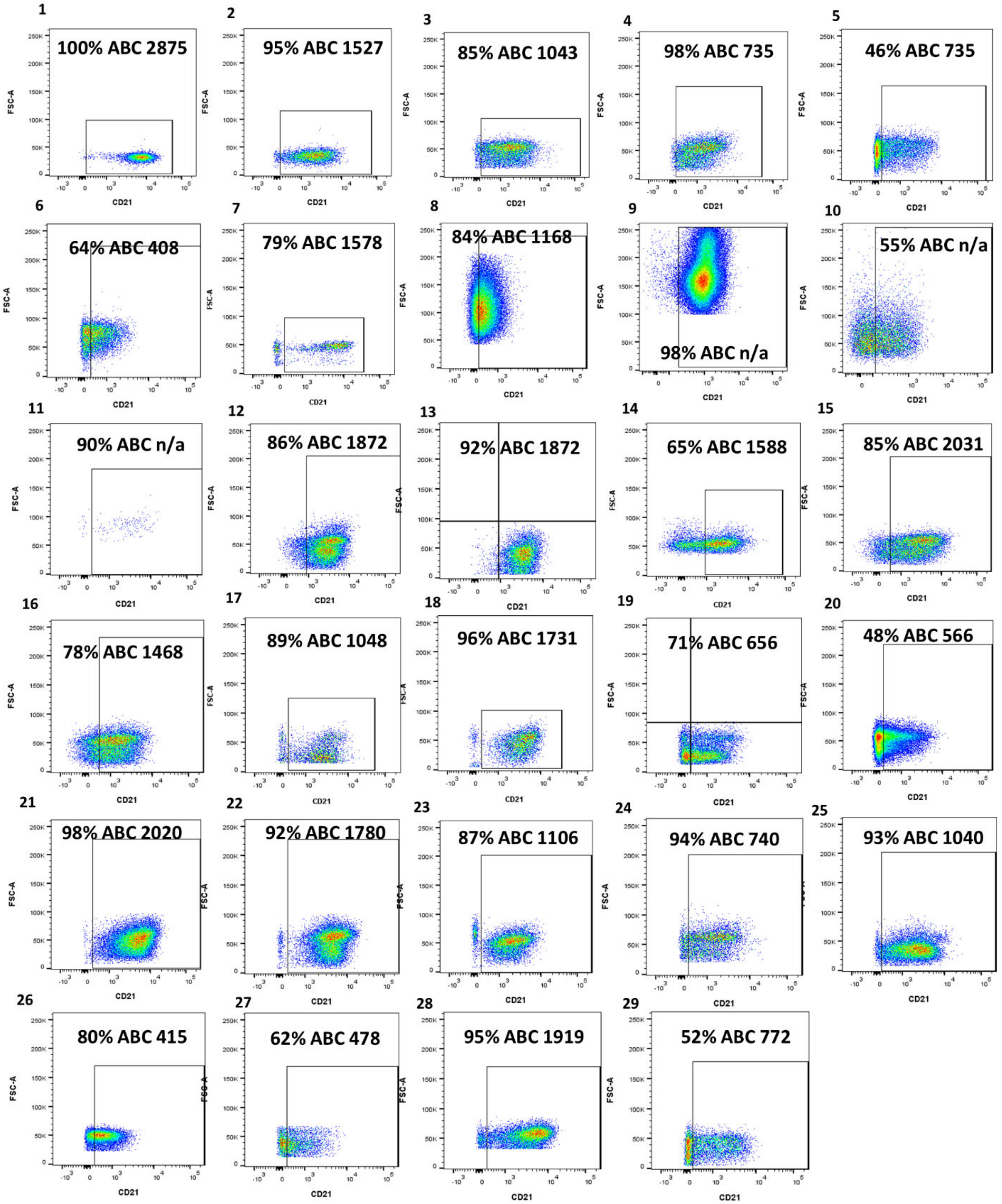

CD21 →

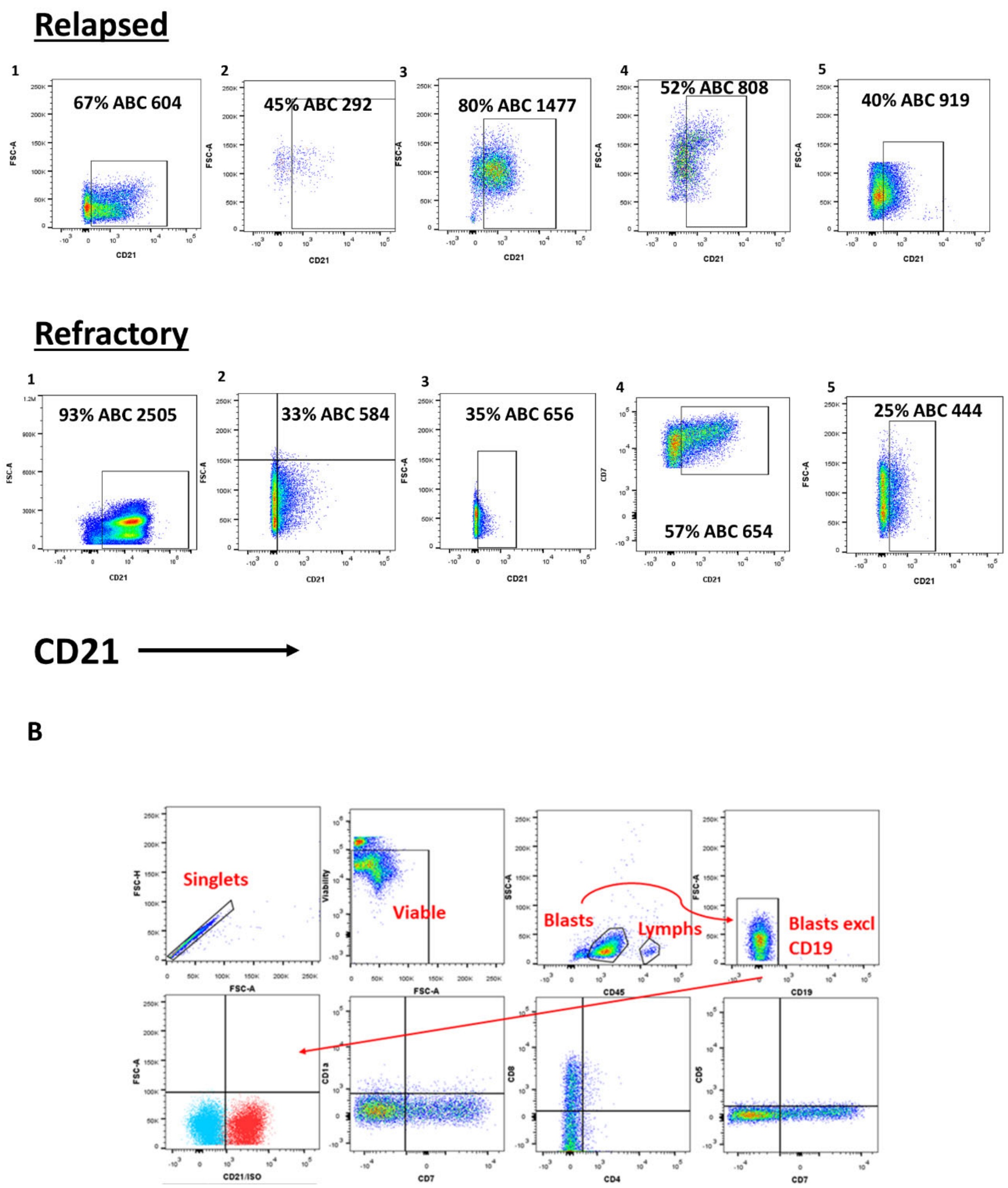

Figure S3: (**A**) Primary flow plots of all CD21 positive diagnostic (n=29), relapsed (n=5) and refractory (n=6) T-ALL patient samples. Percentage of lymphoblasts positive for CD21and CD21 ABC (antibodies bound per cell) listed. Drawn gates based on isotype control (IC) antibody or unstained samples were IC unavailable. (**B**) Example gating strategy for identification of CD21 positive T-lymphoblasts

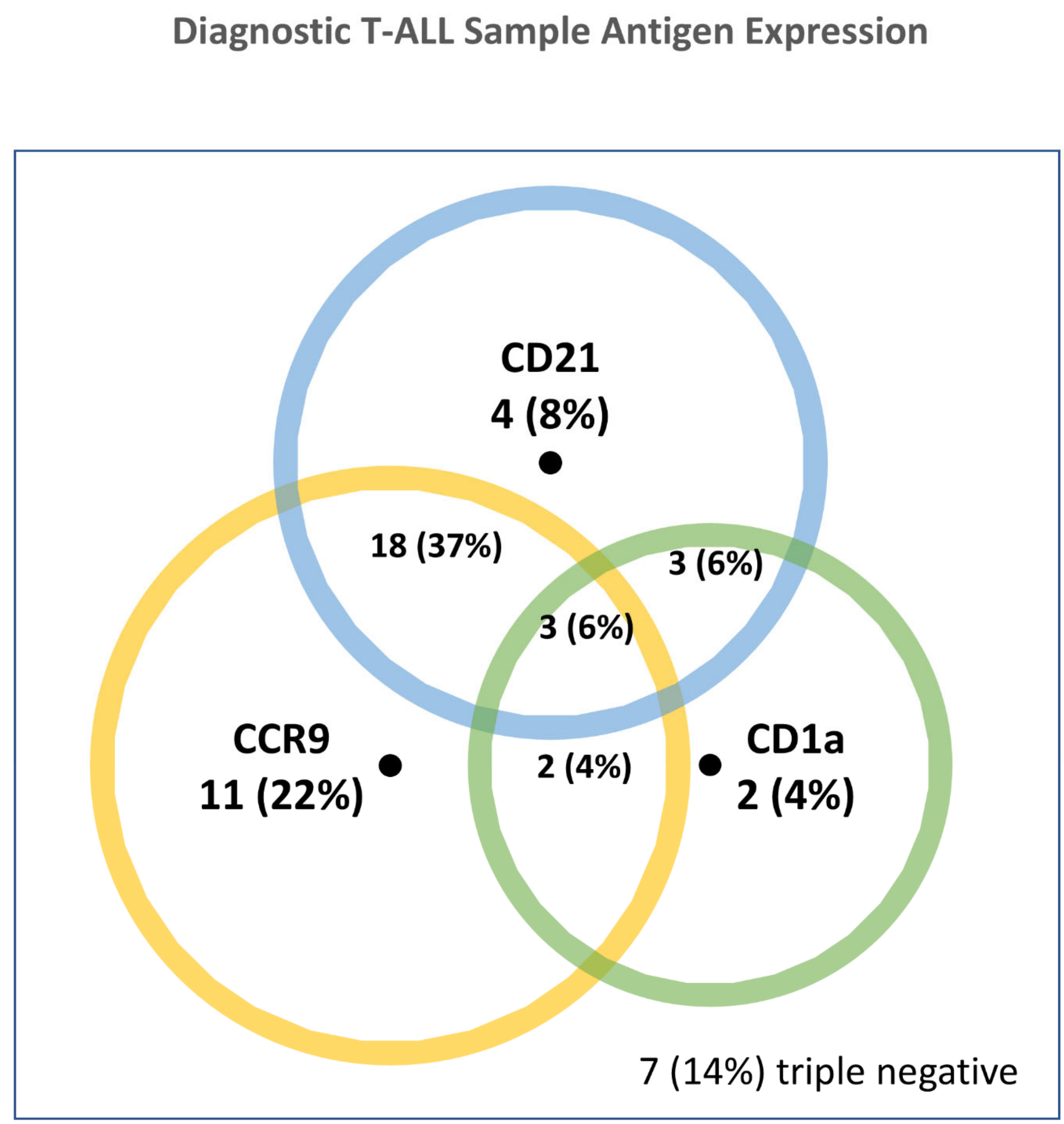

Figure S4: Venn diagram showing overlap expression by flow cytometry of CD21, CCR9 and CD1a in diagnostic T-ALL samples (n=50 cases)

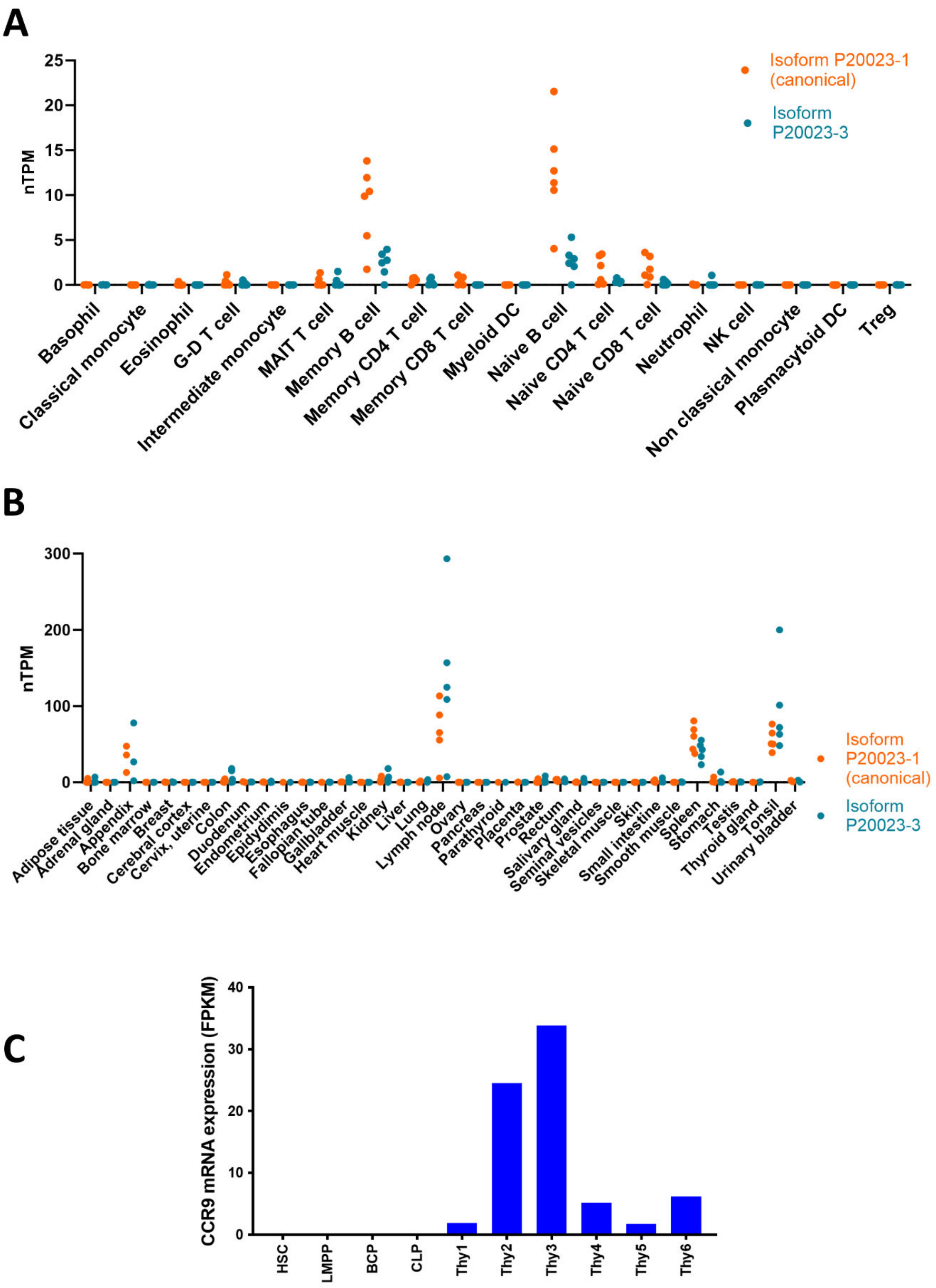

Figure S5: *CR2* RNA isoform expression on (**A**) hemopoietic and (**B**) normal human tissues. Data taken from the Human Protein Atlas. (**C**) RNA expression of *CR2* in thymic subsets. Data taken from a large published dataset (Casero et al 2015).

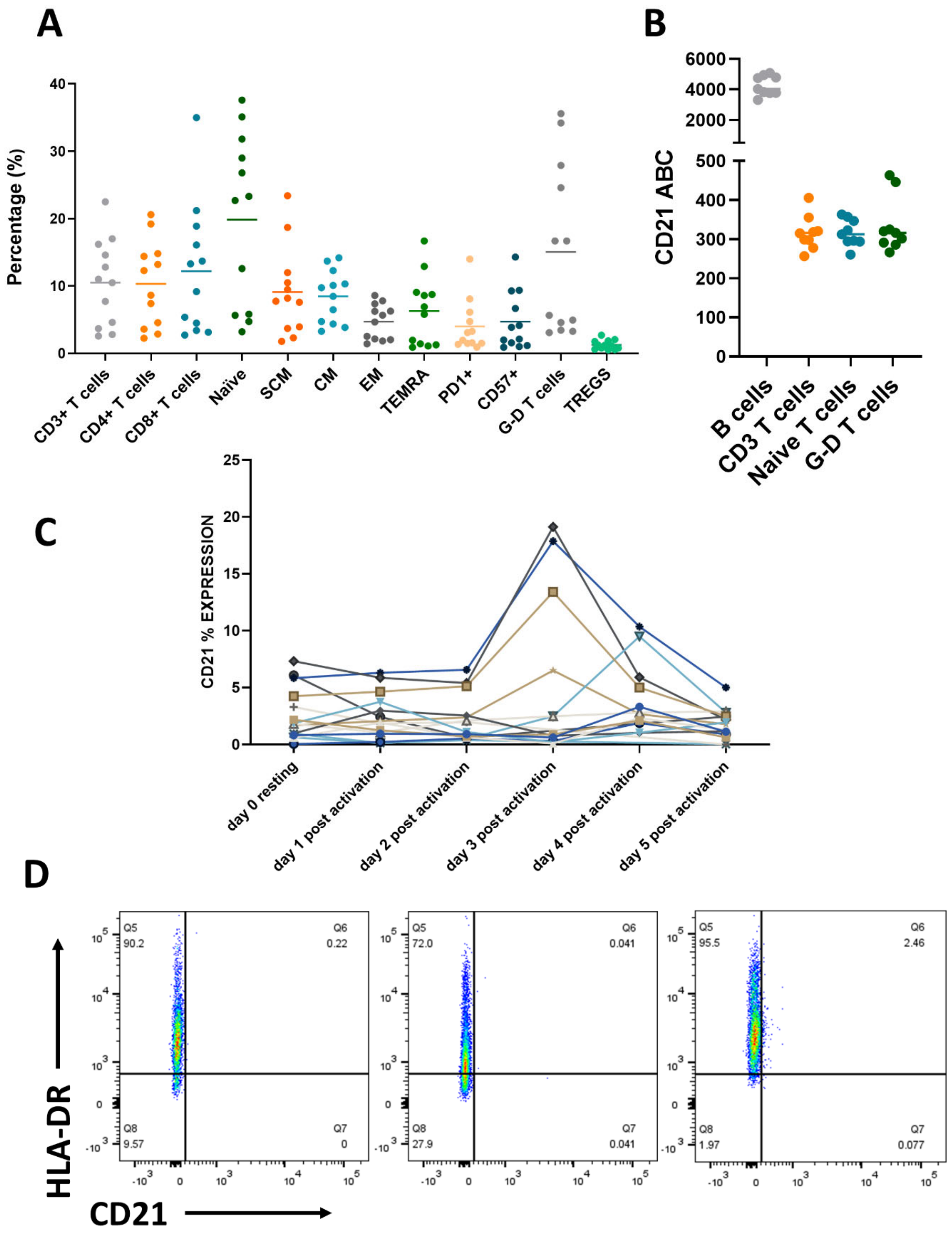

Figure S6: (**A**) Immunophenotypic analysis of CD21 expression on T cell subsets. Data represents three experimental repeats, n=12 healthy donors. (**B**) CD21 ABC (antibodies bound per cell) on T cell subsets. (**C**) CD21 expression by flow cytometry on healthy donor resting

T cells and at set timepoints following T cell activation. 3 experimental repeats, n=15 donors. (**D**) Example flow plots of three healthy donors showing T cell CD21 expression versus HLA-DR expression.

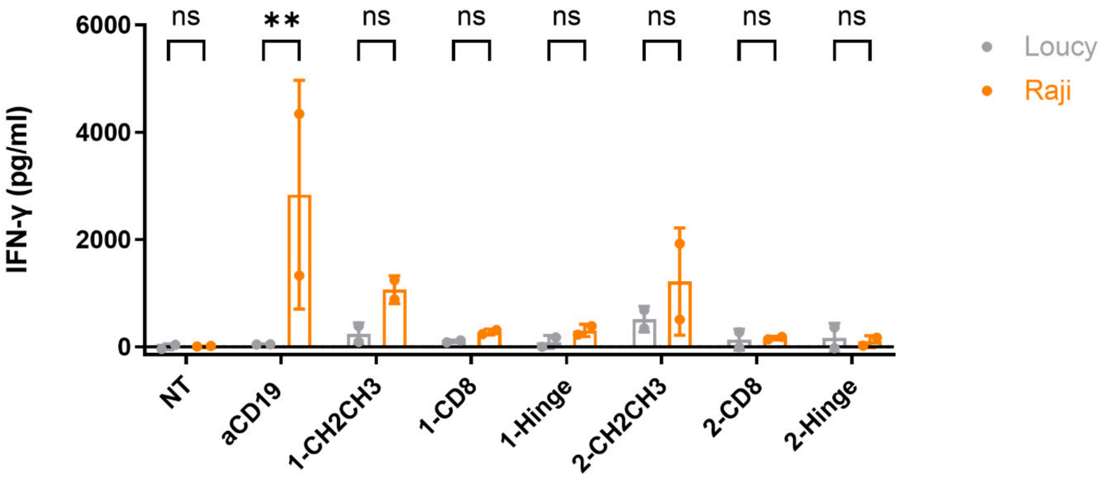

Figure S7: IFN- γ release from CARs containing three spacer regions targeting full length CD21. Data represents IFN-γ in cellular supernatant after CARs were co-cultured with target cell lines for 48hrs in a 1:1 E:T ratio. CAR 1 = scFv C43. CAR 2 = scFv C48. CH2CH3 = 'long' spacer, CD8 = 'medium' spacer, hinge = 'short' spacer. Loucy = CD21 negative. Raji = CD21 positive. No significant difference was seen when groups were compared using 2-way ANOVA. ***P*=0.0061, ns = > 0.05

A

Binder	ka (1/Ms)	kd (1/s)	KD (M)
Bu32	2.74E+05	6.74E-05	2.46E-10
Ph1	1.79E+05	1.52E-04	8.47E-10
Ph17	1.31E+05	5.11E-04	3.91E-09
Ph15	1.45E+05	1.10E-03	7.60E-09
Ph6	8.42E+05	6.54E-03	7.77E-09
Ph12	5.58E+04	4.69E-04	8.41E-09
Ph14	2.44E+05	3.02E-03	1.24E-08
Ph11	8.24E+04	1.43E-03	1.74E-08
Ph5	9.19E+04	1.82E-03	1.98E-08
Ph10	1.20E+05	3.01E-03	2.50E-08
Ph3	9.79E+04	2.52E-03	2.58E-08
Ph7	3.96E+04	1.74E-03	4.40E-08
Ph18	7.74E+04	4.65E-03	6.01E-08
Ph9	1.88E+05	1.24E-02	6.59E-08
NM2	5.21E+05	2.31E-02	8.42E-08
NM1	7.51E+04	1.66E-03	1.01E-06

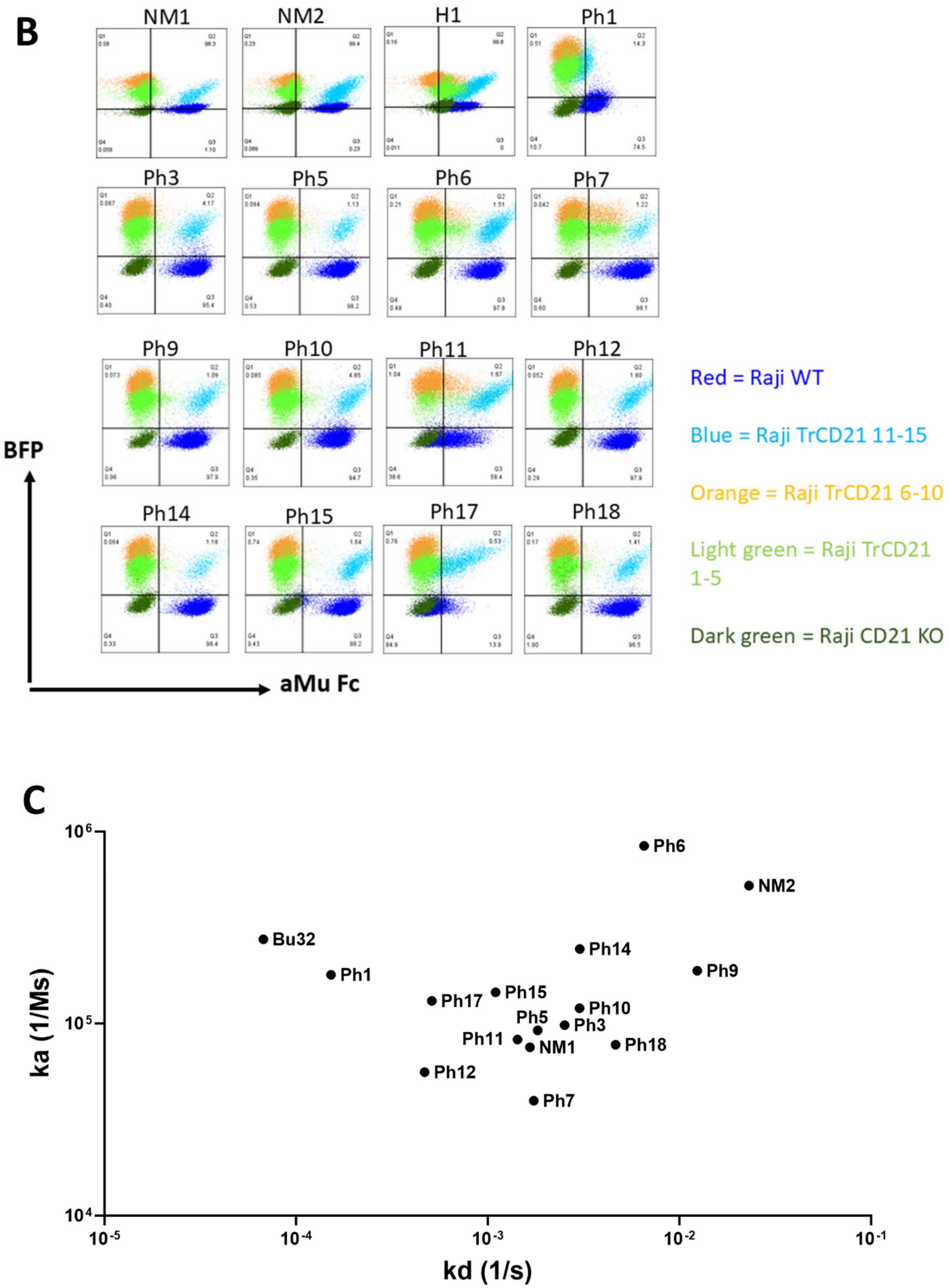
B
NM1
NM2
H1
Ph1
Ph3
Ph5
Ph6
Ph7
Ph9
Ph10
Ph11
Ph12
Ph14
Ph15
Ph17
Ph18
BFP
aMu Fc
Red = Raji WT
Blue = Raji TrCD21 11-15
Orange = Raji TrCD21 6-10
Light green = Raji TrCD21 1-5
Dark green = Raji CD21 KO
C
ka (1/Ms)
kd (1/s)
Ph6
NM2
Bu32
Ph1
Ph14
Ph9
Ph17
Ph15
Ph10
Ph5
Ph3
Ph11
NM1
Ph18
Ph12
Ph7

Figure S8: (**A**) Surface plasmon resonance (SPR) affinity measurements for scFvs binding to CD21 membrane-proximal epitopes, listed by KD. (**B**) Epitope mapping of membrane-proximal binding CD21 scFvs. Raji cell lines engineered to express CD21 truncations contained a BFP marker gene. Dark green = Raji21KO, Light green = Raji 21 SCR1-5, Orange = Raji 21 SCR6-10, Dark blue = Raji WT, Light blue = Raji21 SCR11-15. BFP = blue fluorescent protein. (**C**) On versus off rates for membrane-proximal binding CD21 scFvs. Bu32 = commercial anti-CD21 antibody clone. H1 scFv from hybridoma campaign was unable to be purified and so SPR measurements are not available for this scFv. kon (ka) = association rate constant, koff (kd) = dissociation rate constant, KD (kd/ka) = equilibrium dissociation constant.

	VH ID	Binds to CD21	Cytotoxicity	Basal IFN release
**Ph9**	**V8-22*01 F**	Y	Y	Y
**Ph10**	**V8-22*01 F**	Y	Y	Y
**Ph12**	**V8S18*01 F**	Y	N	Y
**Ph17**	**V8-22*01 F**	N	N	Y
**Ph18**	**V8-22*01 F**	Y	N	Y
**NM2**	**V8-22*01 F**	Y	Y	Y
**Ph3**	**V5-7*01 F**	Y	N	N
**Ph4**	**V5-7*01 F**	N	not tested	not tested
**Ph5**	**V5-7*01 F**	Y	N	N
**Ph14**	**V5-7*01 F**	Y	N	N
**NM1**	**V5-7*01 F**	Y	N	N
**Ph1**	**V8-31*01 F**	Y	N	N
**Ph2**	**V8-23*01 F**	N	N	N
**Ph6**	**V9-4*01 F**	Y	N	N
**Ph7**	**V1-49*01 ORF**	Y	N	N
**Ph11**	**V1-43*01 F**	Y	N	N
**Ph15**	**V1-11*01 F**	Y	N	Y
**H1**	**V2S12*01 F**	Y	N	N
**Bu32**	**Murine derived**	Y	N	Y

Figure S9: All identified CD21 scFvs binding to proximal CD21 epitopes arranged by heavy chain CDR3. Comparison of CD21 binding, CD21 specific cytotoxicity and basal IFN- γ release between scFvs.

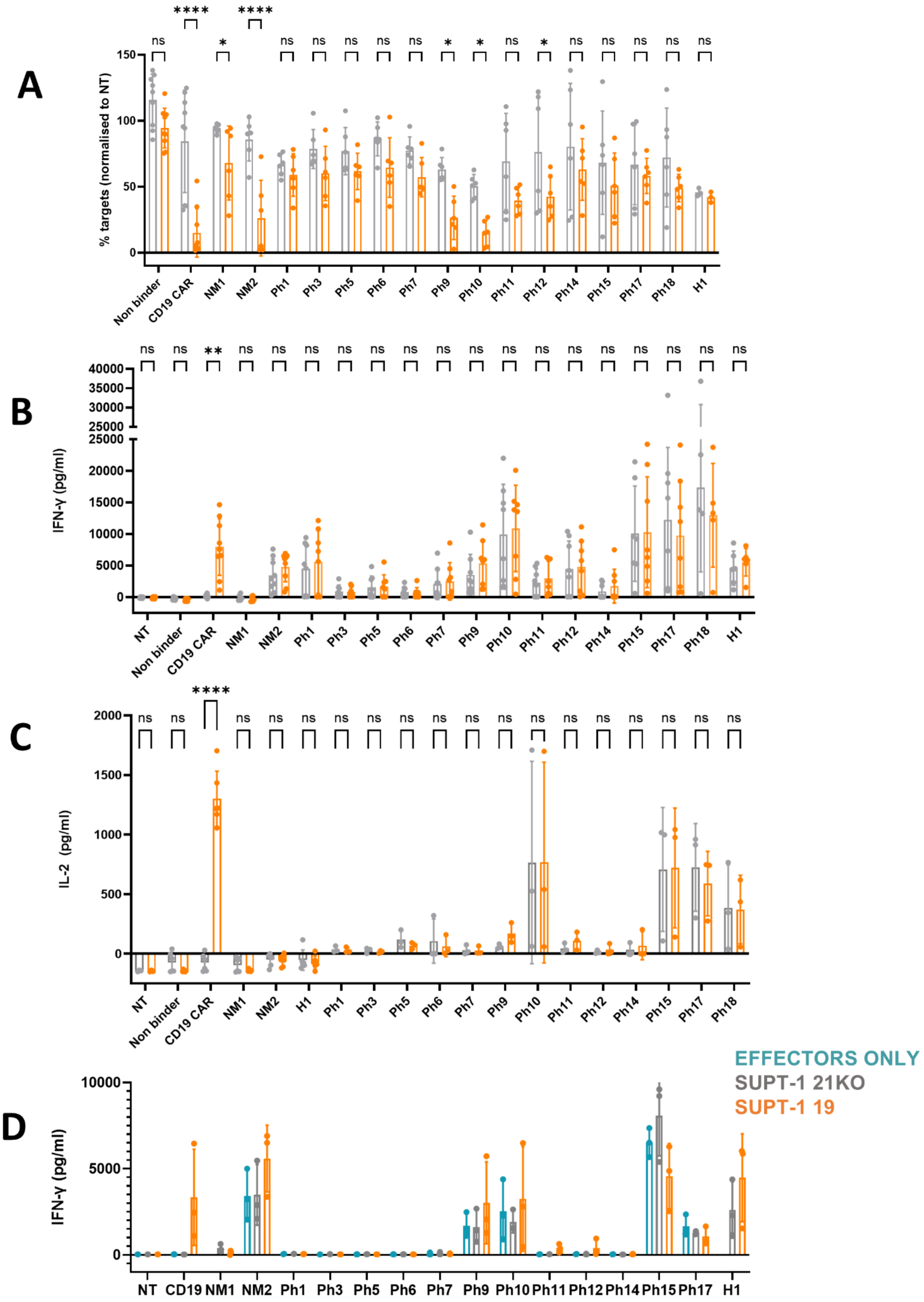

A
% targets (normalised to NT)
150
100
50
0
ns
****
*
****
ns
ns
ns
ns
ns
*
*
ns
*
ns
ns
ns
ns
ns
Non binder
CD19 CAR
NM1
NM2
Ph1
Ph3
Ph5
Ph6
Ph7
Ph9
Ph10
Ph11
Ph12
Ph14
Ph15
Ph17
Ph18
H1
B
IFN-γ (pg/ml)
40000
35000
30000
25000
25000
20000
15000
10000
5000
0
ns
ns
**
ns
ns
ns
ns
ns
ns
ns
ns
ns
ns
ns
ns
ns
ns
ns
ns
NT
Non binder
CD19 CAR
NM1
NM2
Ph1
Ph3
Ph5
Ph6
Ph7
Ph9
Ph10
Ph11
Ph12
Ph14
Ph15
Ph17
Ph18
H1
C
IL-2 (pg/ml)
2000
1500
1000
500
0
ns
ns
****
ns
ns
ns
ns
ns
ns
ns
ns
ns
ns
ns
ns
ns
ns
ns
ns
NT
Non binder
CD19 CAR
NM1
NM2
H1
Ph1
Ph3
Ph5
Ph6
Ph7
Ph9
Ph10
Ph11
Ph12
Ph14
Ph15
Ph17
Ph18
EFFECTORS ONLY
SUPT-1 21KO
SUPT-1 19
D
IFN-γ (pg/ml)
10000
5000
0
NT
CD19
NM1
NM2
Ph1
Ph3
Ph5
Ph6
Ph7
Ph9
Ph10
Ph11
Ph12
Ph14
Ph15
Ph17
H1

Figure S10: Chimeric Antigen Receptor (CAR)-T cells targeting CD21 membrane proximal epitopes display enhanced *in vitro* function (**A**) Summary data of cytotoxicity of non-binding aCD21 CAR (negative control), aCD19 CAR (positive control) and all CD21 membrane-proximal binding CAR-T cells against Supt-1 21KO (grey, CD19/21 neg) and Supt-1 19 (orange, CD19/21 pos) targets after 48hrs by flow cytometry. E:T 1:4, n=3-9 from multiple experiments. Comparisons made by 2-way ANOVA. *P = <0.05, ****P = <0.0001, ns = >0.05 (**B**) Summary data of IFN-γ secretion as determined by ELISA on 1:4 E:T culture supernatant by NT, non-binding aCD21 CAR, anti-CD19 and anti-CD21 CAR-T cells against SUPT-1 19 and SUPT-1 21KO after 48hr co-culture. n=6-9 healthy donors from multiple experiments. Comparisons made by 2-way ANOVA. **P = <0.01 (**C**) Summary data of IL-2 secretion as determined by ELISA on 1:4 E:T culture supernatant by NT, non-binding aCD21 CAR, anti-CD19 and anti-CD21 CAR-T cells against SUPT-1 19 and SUPT-1 21KO after 48hr co-culture. n=6-9 healthy donors from multiple experiments. Comparisons made by 2-way ANOVA. *P = <0.05, ****P = <0.0001. (**D**) IFN-γ secretion as determined by ELISA on 1:4 E:T culture supernatant by NT, anti-CD19 and anti-CD21 CAR-T cells when plated without targets (effectors alone) as well as against SUPT-1 19 and SUPT-1 21KO after 48hr co-culture. n=3 healthy donors from one experiment.

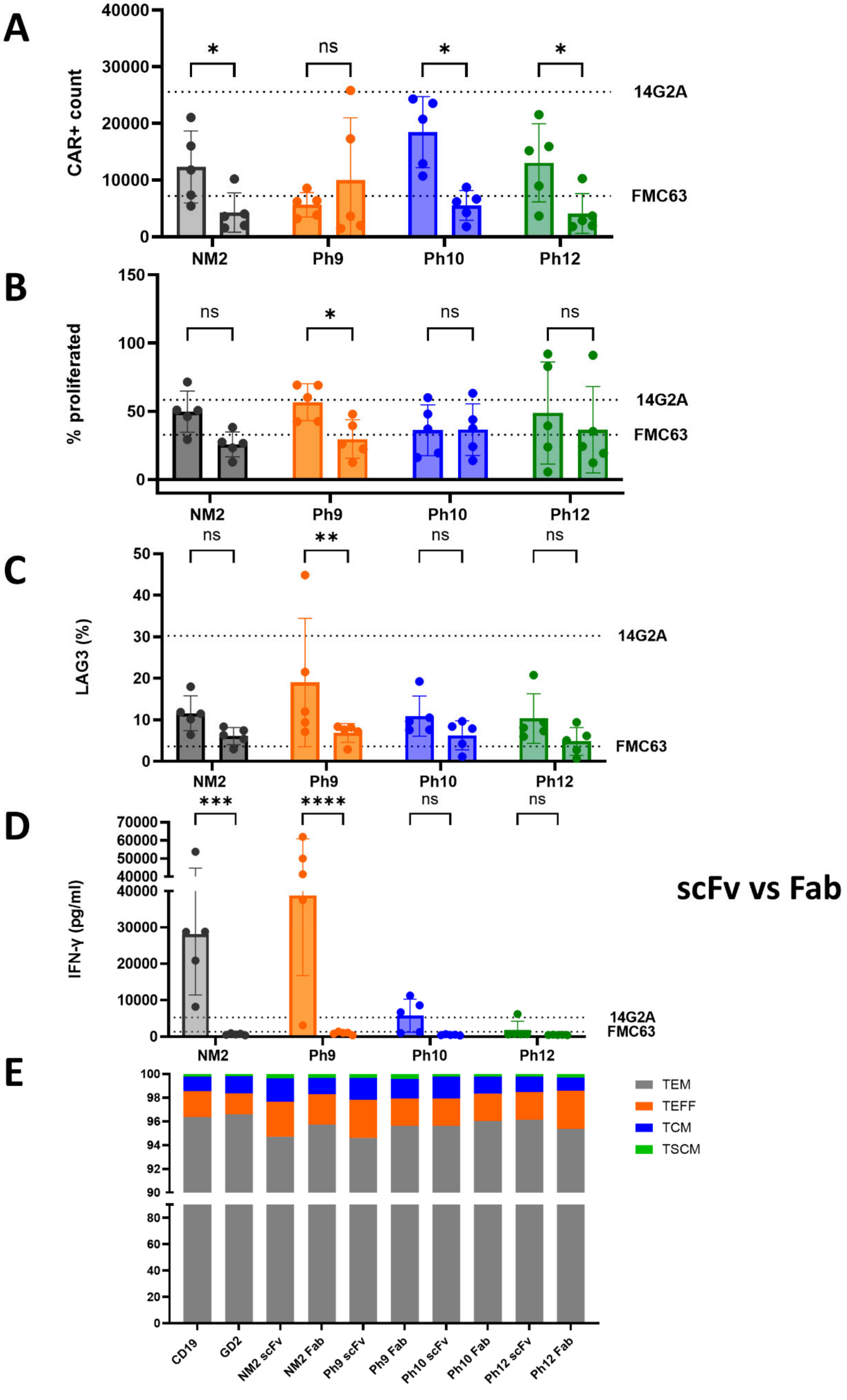
A
CAR+ count
40000
30000
20000
10000
0
*
ns
*
*
14G2A
FMC63
NM2
Ph9
Ph10
Ph12
B
% proliferated
150
100
50
0
ns
*
ns
ns
14G2A
FMC63
NM2
Ph9
Ph10
Ph12
C
LAG3 (%)
50
40
30
20
10
0
ns
**
ns
ns
14G2A
FMC63
NM2
Ph9
Ph10
Ph12
D
IFN-γ (pg/ml)
70000
60000
50000
40000
40000
30000
20000
10000
0
***
****
ns
ns
14G2A
FMC63
NM2
Ph9
Ph10
Ph12
scFv vs Fab
E
100
98
96
94
92
90
80
60
40
20
0
TEM
TEFF
TCM
TSCM
CD19
GD2
NM2 scFv
NM2 Fab
Ph9 scFv
Ph9 Fab
Ph10 scFv
Ph10 Fab
Ph12 scFv
Ph12 Fab

Figure S11: (**A**) Cell count, (**B**) % proliferation, (**C**) % LAG3 expression and (**D**) IFN-γ secretion by NM2, Ph9, Ph10 and Ph12 aCD21 scFv (left) versus Fab (right) CAR-T cells on day 7 after plating without targets or exogenous cytokines. Dotted lines = positive (14G2A) and negative (FMC63) control. N=5. (**E**) Memory T cell subsets between scFv and Fab based CARs on day 7. TSCM = stem cell memory T cells, TCM = central memory T cells, TEM = effector memory T cells, TEMRA = terminally differentiated effector memory T cells. * *P* = <0.05. ** *P* = <0.01. *** *P* = < 0.001 **** *P* = <0.0001, ns = not significant *P* = >0.05.

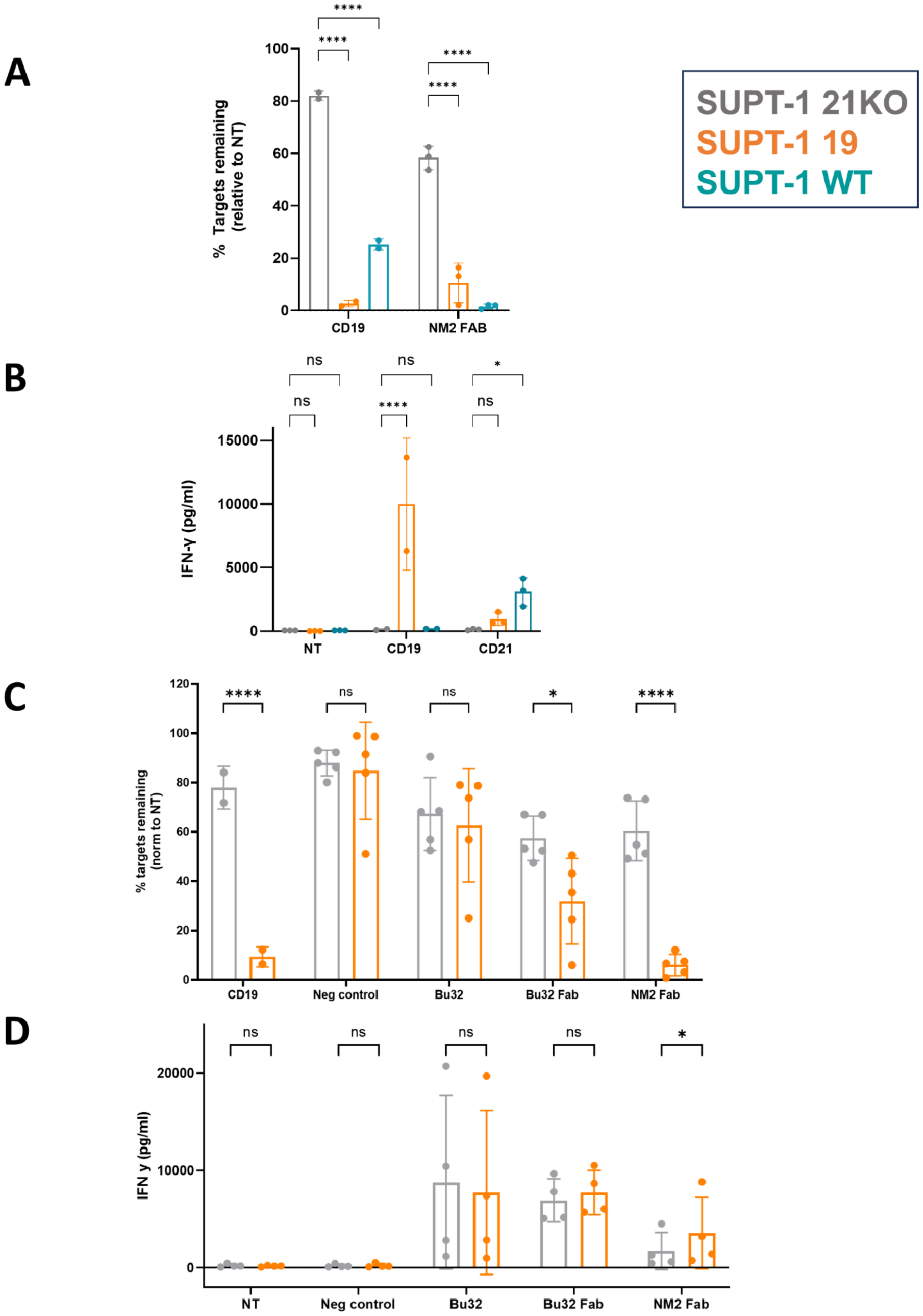
A
B
C
D
SUPT-1 21KO
SUPT-1 19
SUPT-1 WT
% Targets remaining (relative to NT)
CD19
NM2 FAB
****
IFN-γ (pg/ml)
NT
CD19
CD21
ns
*
% targets remaining (norm to NT)
CD19
Neg control
Bu32
Bu32 Fab
NM2 Fab
IFN y (pg/ml)
NT
Neg control
Bu32
Bu32 Fab
NM2 Fab

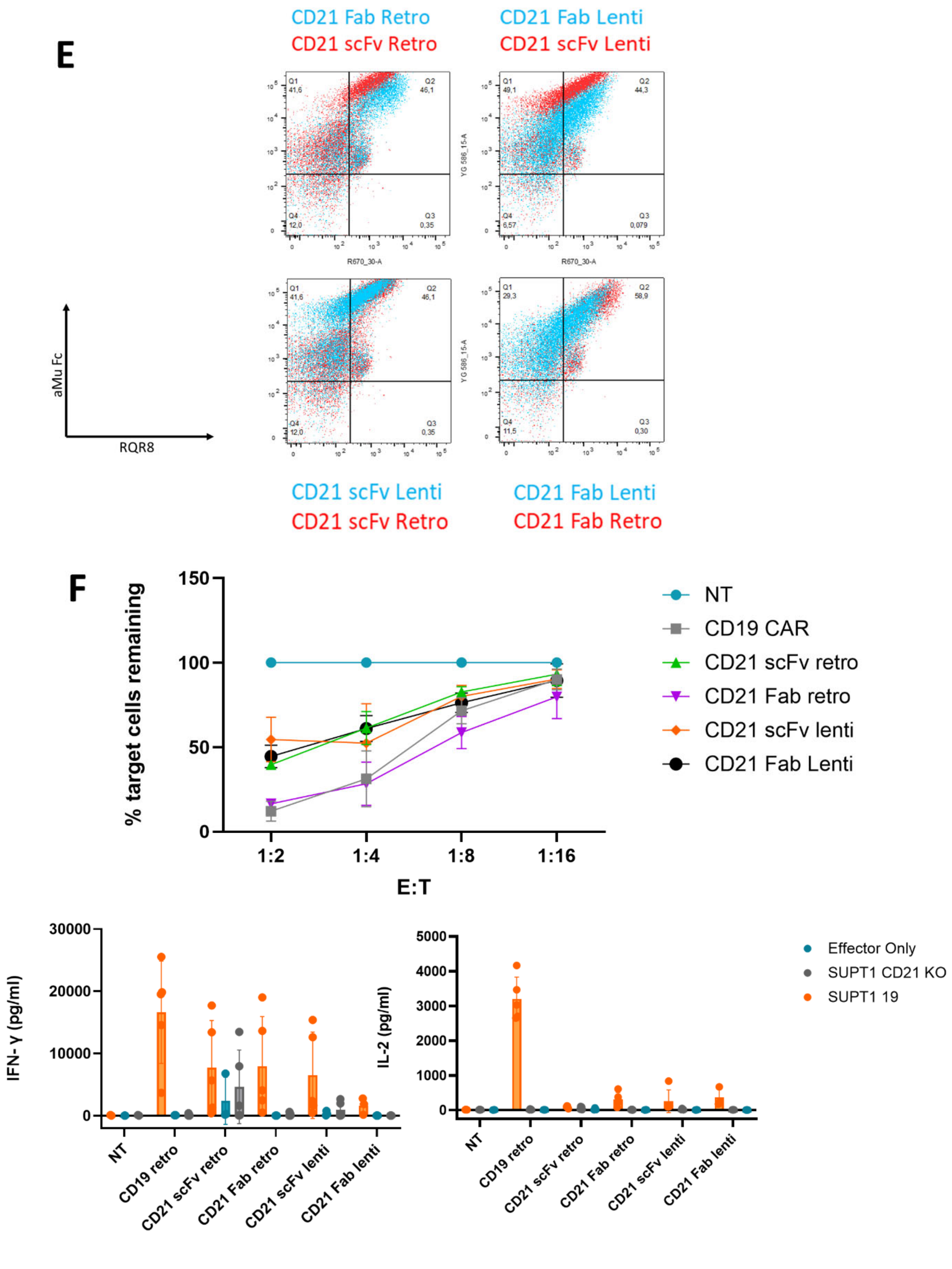

Figure S12: (**A**) Cytotoxicity and (**B**) IFN-γ release of aCD21 NM2 Fab CAR in 48hr co-culture against SUPT1 WT (21+/19-), SUPT1-19 (21+/19+) and SUPT1 21KO (21-/19-). n=3 donors, one experiment. E:T ratio 1:4. Comparisons made with 2-way ANOVA. Ns = >0.05

P* = <0.05 *****P* = <0.0001 (C-D**) aCD21 CAR-T cells – NM2 Fab, Bu32, Bu32 Fab were co-cultured with target cell lines (grey = SUPT121KO, orange = SUPT1 19) for 48hrs. CD19 CAR = positive control. Ph2 nonbinding CAR = negative control. (**C**) % target cells remaining at a 1:8 E:T ratio across all CARs. (**D**) IFN-γ release by ELISA on 1:8 E:T ratio 48hr culture supernatant. Summary data of 4-5 healthy donors across two experiments. Graphs show mean plus SD. Comparisons made using 2-way ANOVA. ns = >0.05 **P* = <0.05 *****P* = <0.0001 (**E**) NM2 CD21 CAR expression on the surface of healthy donor T cells (n=1). Comparison of scFv and Fab antigen binding domains and lentiviral and retroviral expression cassettes. (**F**) Cytotoxicity, IFN-γ and IL-2 release by NM2 scFv and Fab-CARs in both retroviral and lentiviral expression cassettes when tested in a 48hr co-culture. Summary results of up to 5 normal donors across three experimental repeats. E:T ratio = 1:4.

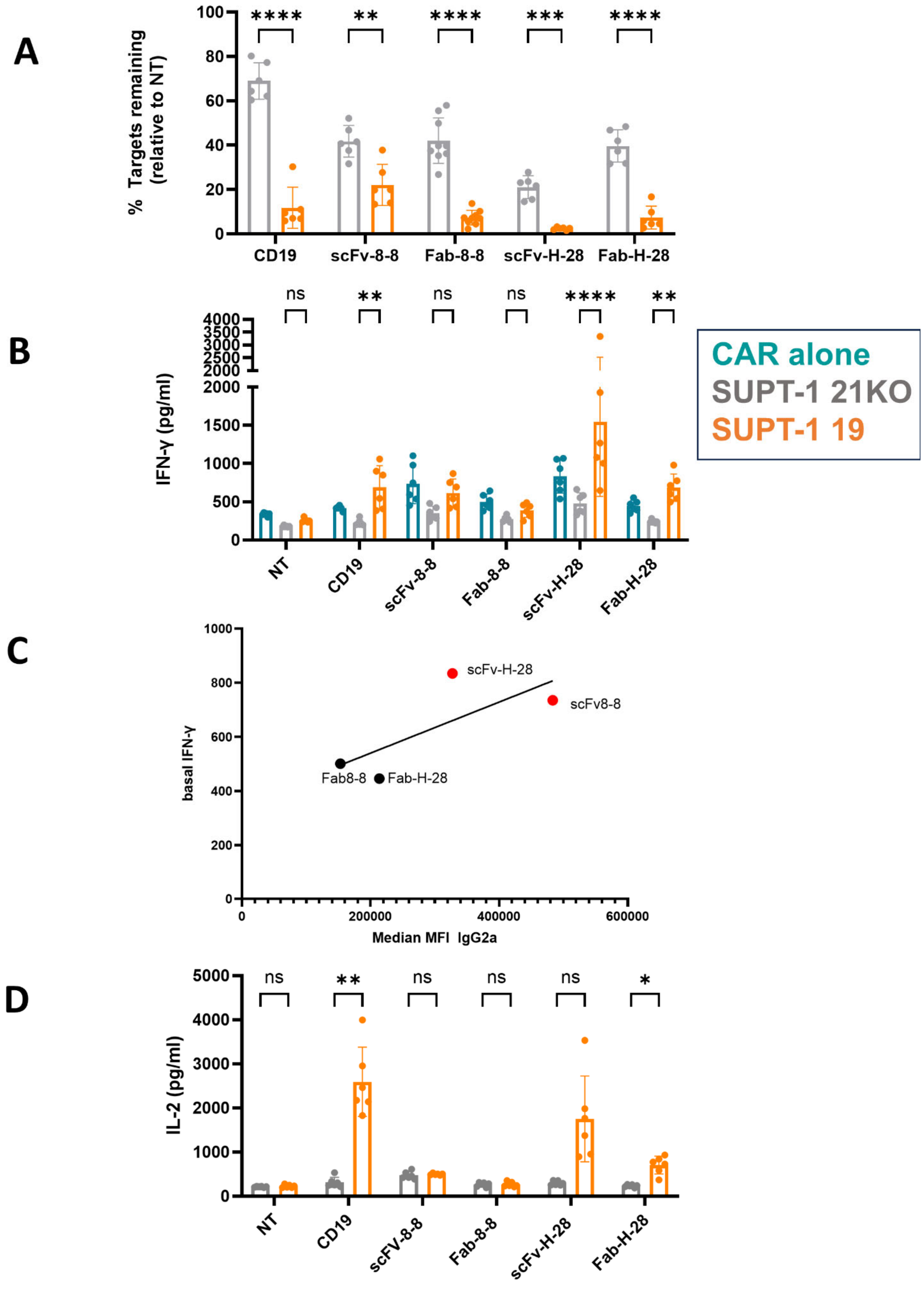
A
% Targets remaining (relative to NT)
CD19
scFv-8-8
Fab-8-8
scFv-H-28
Fab-H-28
****
**
****
***
****
B
IFN-γ (pg/ml)
NT
CD19
scFv-8-8
Fab-8-8
scFv-H-28
Fab-H-28
ns
**
ns
ns
****
**
CAR alone
SUPT-1 21KO
SUPT-1 19
C
basal IFN-γ
Median MFI IgG2a
scFv-H-28
scFv8-8
Fab8-8
Fab-H-28
D
IL-2 (pg/ml)
NT
CD19
scFV-8-8
Fab-8-8
scFv-H-28
Fab-H-28
ns
**
ns
ns
ns
*

Figure S13: (**A**) Cytotoxicity, and (**B**) IFN-γ including CARs alone (turquoise) of NM2 scFv 8-8, Fab-8-8, scFv-H-28 and Fab-H-28 based CARs against SUPT1-19 (orange) and SUPT1-21KO (grey) targets, 1:4 E:T, N=6-9, three experimental repeats. (**C**) Correlation between CAR surface expression and basal IFN-γ secretion without targets by simple linear regression, P = 0.26. (**D**) IL-2 secretion of NM2 scFv 8-8, Fab-8-8, scFv-H-28 and Fab-H-28 based CARs against SUPT1-19 (orange) and SUPT1-21KO (grey) targets, 1:4 E:T, N=6-9, three experimental repeats. * P = <0.05. ** P = <0.01. *** P = < 0.001 **** P = <0.0001, ns = not significant.

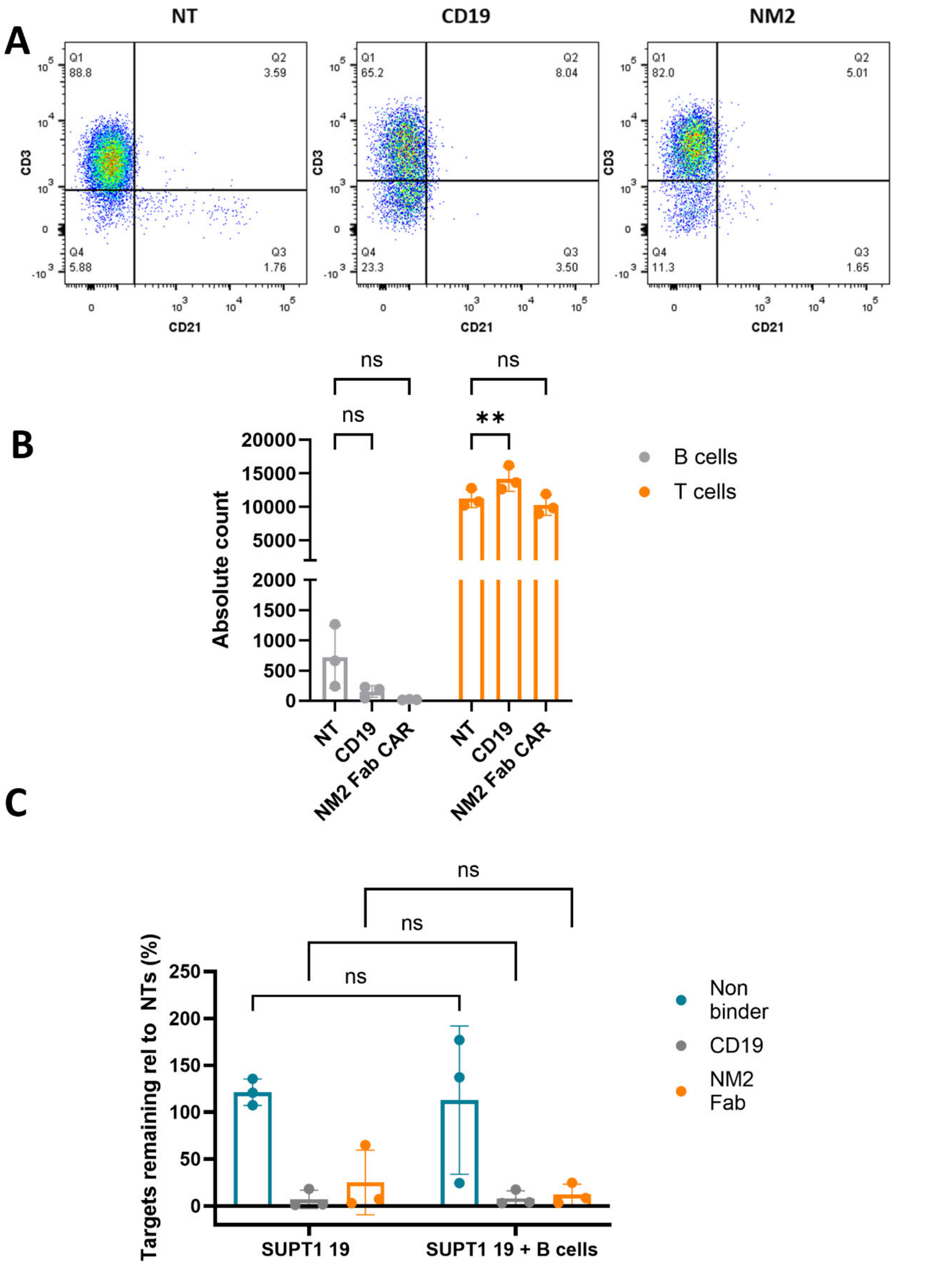

Figure S14: (**A**) CD21 expression on non-transduced (NT) PBMCs and PBMCs post CAR-T transduction with CD19 and NM2 anti-CD21 Fab CARs. Figure shows data from one donor with the same pattern repeated across three donor repeats. (**B**) Residual counts of B and T cells

when non-transduced (NT) T cells, CD19 and NM2 Fab CD21 CAR-T cells were co-cultured for 48hrs against autologous PBMCs. Target PBMCs identified through cell-trace violet (CTV) staining and CD19, CD4/8 staining. (**C**) Cytotoxicity of NM2 Fab aCD21 CAR-T cells when co-cultured against SUPT-19 cells for 48hrs with and without autologous PBMCs 'spiked in' to the co-culture. N=3, one experimental repeat.

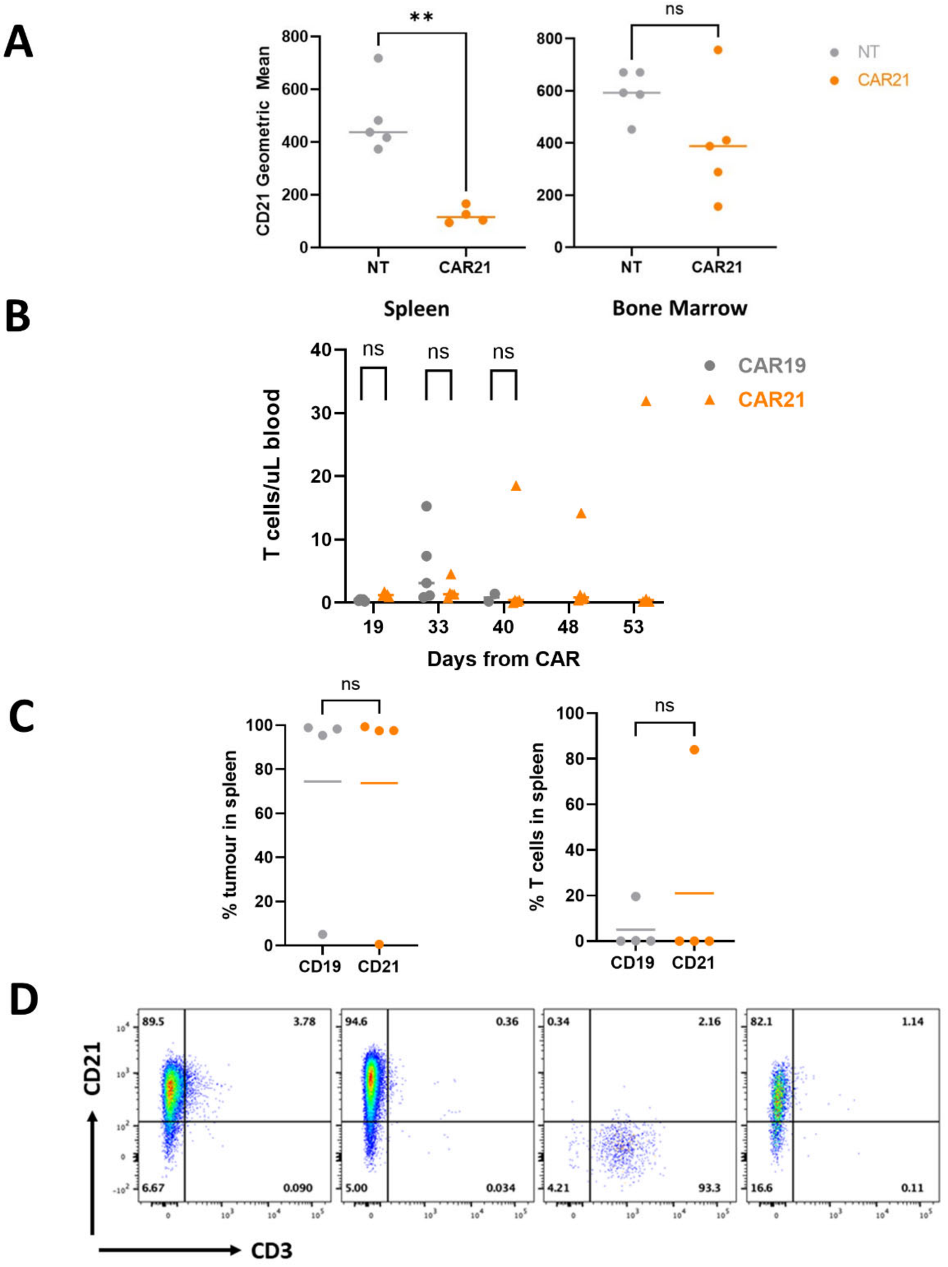

E

Animal	Day of sacrifice	Reason for sacrifice
Control CD19 mouse 1	D+48	High tumor burden
Control CD19 mouse 2	D+45	Weight loss and health concerns
Control CD19 mouse 3	D+48	High tumor burden
Control CD19 mouse 4	D+48	Health concerns ? GVHD
CD21 mouse 1	D+107	Weight loss and health concerns
CD21 mouse 2	D+94	Weight loss and health concerns
CD21 mouse 3	D+123	End of experiment
CD21 mouse 4	D+123	End of experiment

Figure S15: (**A**) Residual tumour by flow cytometry in spleen and bone marrow at necroscopy of NT and CAR21 treated mice engrafted with Jurkat cells. Compared by unpaired t test. Spleen

P = 0.0013, marrow *P* = 0.1085 (**B**) % detectable T cells in blood post CAR for CAR19 (grey) and CAR21 (orange) treated mice in PDX1. Significance assessed by mixed-effects analysis. (**C**) Left: % detectable tumour in spleen and right: % detectable T cells in spleen in CAR19 (grey) and CAR21 (orange) treated mice engrafted with PDX2. Significance assessed by unpaired t test. (**D**) CD21 expression on residual tumour at necroscopy in 4 CAR21 treated mice. (**E**) Individual mouse outcomes in PDX2 experiment.

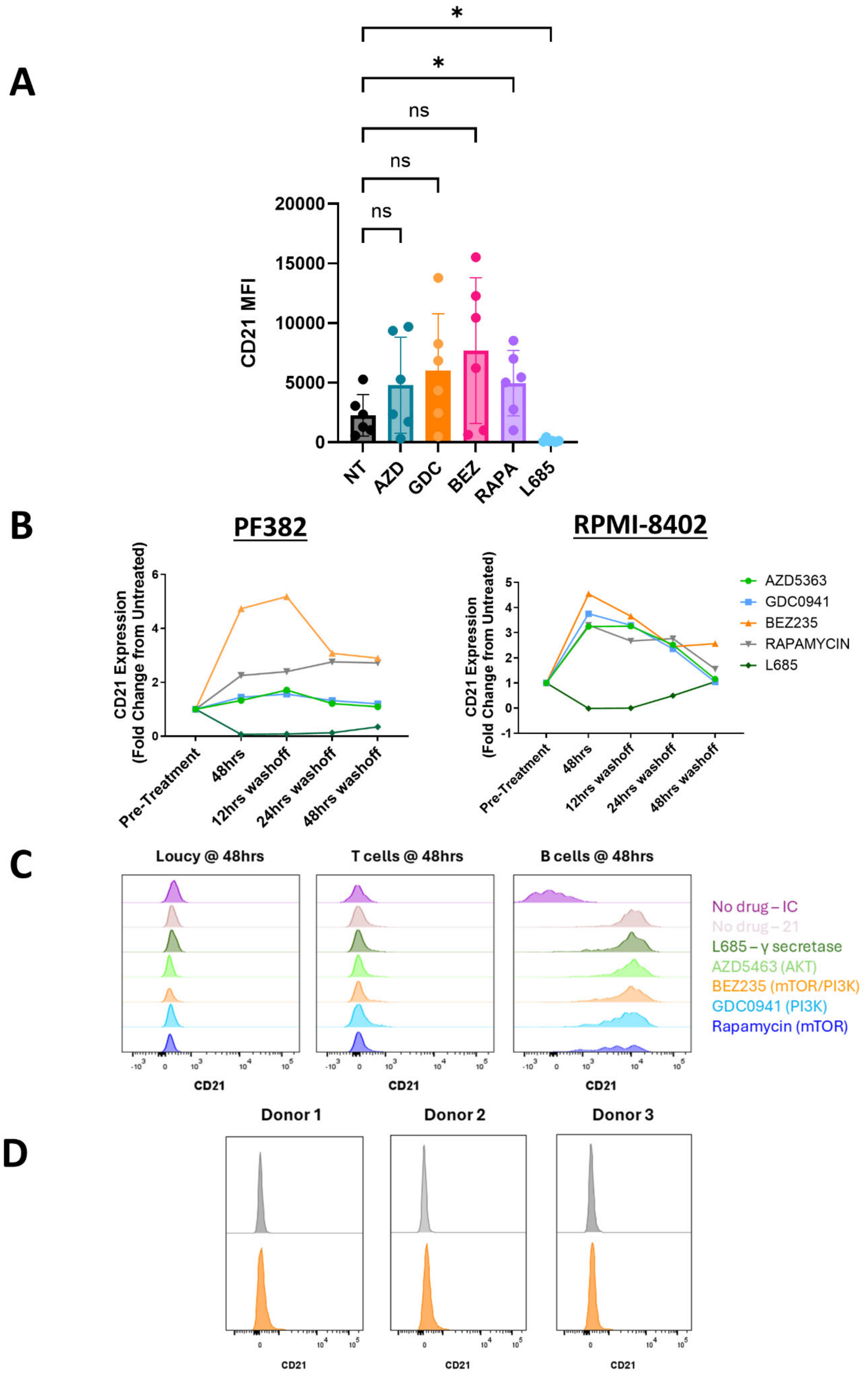
A
B
C
D
CD21 MFI
NT
AZD
GDC
BEZ
RAPA
L685
ns
*
PF382
RPMI-8402
CD21 Expression (Fold Change from Untreated)
Pre-Treatment
48hrs
12hrs washoff
24hrs washoff
48hrs washoff
AZD5363
GDC0941
BEZ235
RAPAMYCIN
L685
Loucy @ 48hrs
T cells @ 48hrs
B cells @ 48hrs
No drug – IC
No drug – 21
L685 – γ secretase
AZD5463 (AKT)
BEZ235 (mTOR/PI3K)
GDC0941 (PI3K)
Rapamycin (mTOR)
CD21
Donor 1
Donor 2
Donor 3

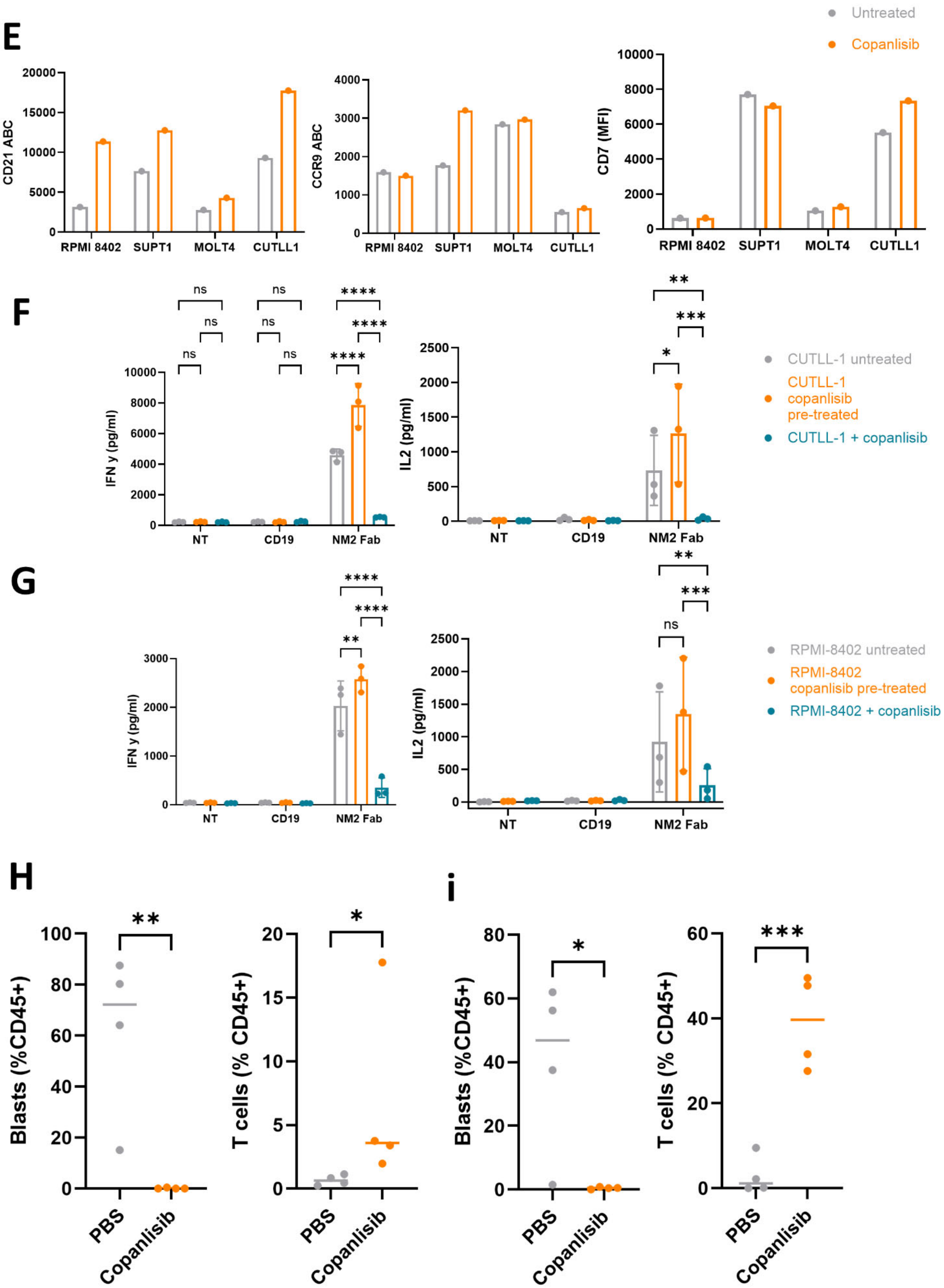
E
Untreated
Copanlisib
CD21 ABC
20000
15000
10000
5000
0
RPMI 8402
SUPT1
MOLT4
CUTLL1
CCR9 ABC
4000
3000
2000
1000
0
RPMI 8402
SUPT1
MOLT4
CUTLL1
CD7 (MFI)
10000
8000
6000
4000
2000
0
RPMI 8402
SUPT1
MOLT4
CUTLL1
F
ns
ns
ns
ns
ns
ns
****
****
****
IFN y (pg/ml)
10000
8000
6000
4000
2000
0
NT
CD19
NM2 Fab
**
***
*
IL2 (pg/ml)
2500
2000
1500
1000
500
0
NT
CD19
NM2 Fab
CUTLL-1 untreated
CUTLL-1 copanlisib pre-treated
CUTLL-1 + copanlisib
G
****
****
**
IFN y (pg/ml)
3000
2000
1000
0
NT
CD19
NM2 Fab
**
***
ns
IL2 (pg/ml)
2500
2000
1500
1000
500
0
NT
CD19
NM2 Fab
RPMI-8402 untreated
RPMI-8402 copanlisib pre-treated
RPMI-8402 + copanlisib
H
**
Blasts (%CD45+)
100
80
60
40
20
0
PBS
Copanlisib
*
T cells (% CD45+)
20
15
10
5
0
PBS
Copanlisib
i
*
Blasts (%CD45+)
80
60
40
20
0
PBS
Copanlisib
***
T cells (% CD45+)
60
40
20
0
PBS
Copanlisib

Figure S16: (**A**) CD21 expression on six T-ALL cell lines (CUTLL-1, PF382, RPMI-8402, SUPT-1, KOPTK1, Jurkat) incubated with PI3K inhibitors (AZD5363, GDC0941, Bez235, and rapamycin) or the gamma-secretase inhibitor L685 at 72hrs after incubation compared to untreated (NT). Data analysed by one-way ANOVA. (**B**) Fold-change in CD21 expression in PF382 and RPMI-8402 at baseline, 48hrs after drug addition, then at 12, 24 and 48hrs after drug removal. (**C**) CD21 expression on T cells, B cells and Loucy cells (NOTCH1 WT) after 48hr incubation with PI3K inhibitors and L685. (**D**) Histograms of HLA-DR +ve activated T cells (n=3) showing CD21 expression by flow cytometry with copanlisib (orange) and without copanlisib (grey). (**E**) T-ALL cell line expression of CD21, CCR9 and CD7 after 48hr incubation of copanlisib. (**F**) IFN-γ and IL-2 secretion by NM2 Fab CAR21 against CUTLL1 untreated, copanlisib pre-treated cells and CUTLL1 cells exposed to copanlisib during co-culture by ELISA on 1:4 E:T 48hr cell culture supernatant. n=3. Comparisons made using 2-way ANOVA. (**G**) IFN-γ and IL-2 secretion by NM2 Fab CAR21 against RPMI-8402 untreated, copanlisib pre-treated cells and RPMI-8402 cells exposed to copanlisib during co-culture by ELISA on 1:4 E:T 48hr cell culture supernatant. n=3. Comparisons made using 2-way ANOVA. (**H**) % bone marrow lymphoblasts and T cells at necroscopy in NSG mice engrafted with T-ALL PDX treated with copanlisib (orange) or PBS (grey) then CAR21 T cells. (**I**) % spleen lymphoblasts and T cells at necroscopy in NSG mice engrafted with T-ALL PDX treated with copanlisib (orange) or PBS (grey) then CAR21 T cells. * *P* =<0.05. ** *P* = <0.01. *** *P* = < 0.001 **** *P* = <0.0001, ns = not significant.

A MGAAGLLGVFLALVAPGVLGISCGSPPPILNGRISYYSTPIAVGTVIRYSCS
GTFRLIGEKSLLCITKDKVDGTWDKPAPKCEYFNKYSSCPEPIVPGGYKIR
GSTPYRHGDSVTFACKTNFSMNGNKSVWCQANNMWGPTRLPTCVSV
FPLECPALPMIHNGHHTSENVGSIAPGLSVTYSCESGYLLVGEKIINCLSSG
KWSAVPPTCEEARCKSLGRFPNGKVKEPPILRVGVTANFFCDEGYRLQG
PPSSRCVIAGQGVAWTKMPVCEEIFCPSPPPILNGRHIGNSLANVSYGSI
VTYTCDPDPEEGVNFILIGESTLRCTVDSQKTGTWSGPAPRCELSTSAVQ
CPHPQILRGRMVSGQKDRYTYNDTVIFACMFGFTLKGSKQIRCNAQGT
WEPSAPVCEKECQAPPNILNGQKEDRHMVRFDPGTSIKYSCNPGYVLV
GEESIQCTSEGVWTPPVPQCKVAACEATGRQLLTKPQHQFVRPDVNSS
CGEGYKLSGSVYQECQGTIPWFMEIRLCKEITCPPPPVIYNGAHTGSSLE
DFPYGTTVTYTCNPGPERGVEFSLIGESTIRCTSNDQERGTWSGPAPLCK
LSLLAVQCSHVHIANGYKISGKEAPYFYNDTVTFKCYSGFTLKGSSQIRCK
ADNTWDPEIPVCEKETCQHVRQSLQELPAGSRVELVNTSCQDGYQLTG
HAYQMCQDAENGIWFKKIPLCKVIHCHPPPVIVNGKHTGMMAENFLY
GNEVSYECDQGFYLLGEKKLQCRSDSKGHGSWSGPSPQCLRSPPVTRCP
NPEVKHGYKLNKTHSAYSHNDIVYVDCNPGFIMNGSRVIRCHTDNTWV
PGVPTCIKKAFIGCPPPPKTPNGNHTGGNIARFSPGMSILYSCDQGYLLV
GEALLLCTHEGTWSQPAPHCKEVNCSSPADMDGIQKGLEPRKMYQYG
AVVTLECEDGYMLEGSPQSQCQSDHQWNPPLAVCRSRSLAPVLCGIAA
GLILLTFLIVITLYVISKHRARNYYTDTSQKE

B MGAAGLLGVFLALVAPGVLGETCQHVRQSLQELPAGSRVELVNTSCQD
GYQLTGHAYQMCQDAENGIWFKKIPLCKVIHCHPPPVIVNGKHTGMMA
ENFLYGNEVSYECDQGFYLLGEKKLQCRSDSKGHGSWSGPSPQCLRSPP
VTRCPNPEVKHGYKLNKTHSAYSHNDIVYVDCNPGFIMNGSRVIRCHTD
NTWVPGVPTCIKKAFIGCPPPPKTPNGNHTGGNIARFSPGMSILYSCDQ
GYLLVGEALLLCTHEGTWSQPAPHCKEVNCSSPADMDGIQKGLEPRKMY
QYGAVVTLECEDGYMLEGSPQSQCQSDHQWNPPLAVCRSRSLAPVLCG
IAAGLILLTFLIVITLYVISKHRARNYYTDTSQKE

Figure S17: (**A**) Amino acid sequence of CD21 ectodomain, transmembrane and truncated endodomain in rat vaccination campaign (Uniprot P20023-1 AA sequence 1-1014) (**B**) AA sequence of SCRs 11-15 of CD21 ectodomain with a truncated endodomain used in rat vaccination campaign (Uniprot P20023-1 AA sequence 660-1014)
